# Characterization of Polymer Degrading Lipases, LIP1 and LIP2 From *Pseudomonas chlororaphis* PA23

**DOI:** 10.3389/fbioe.2022.854298

**Published:** 2022-04-20

**Authors:** Nisha Mohanan, Chun Hin Wong, Nediljko Budisa, David B. Levin

**Affiliations:** ^1^ Department of Biosystems Engineering, University of Manitoba, Winnipeg, MB, Canada; ^2^ Department of Chemistry, University of Manitoba, Winnipeg, MB, Canada

**Keywords:** lipase, esterase, polyhydroxyalkanoate (PHA), polylactic acid (PLA), poly(εcaprolactone) (PCL), polyethylene succinate (PES), biodegradation

## Abstract

The outstanding metabolic and bioprotective properties of the bacterial genus *Pseudomonas* make these species a potentially interesting source for the search of hydrolytic activities that could be useful for the degradation of plastics. We identified two genes encoding the intracellular lipases LIP1 and LIP2 of the biocontrol bacterium *Pseudomonas chlororaphis* PA23 and subsequently performed cloning and expression in *Escherichia coli*. The *lip1* gene has an open reading frame of 828 bp and encodes a protein of 29.7 kDa whereas the *lip2* consists of 834 bp and has a protein of 30.2 kDa. Although secondary structure analyses of LIP1 and LIP2 indicate a dominant α/β-hydrolase-fold, the two proteins differ widely in their amino acid sequences (15.39% identity), substrate specificities, and hydrolysis rates. Homology modeling indicates the catalytic serine in both enzymes located in a GXSXG sequence motif (lipase box). However, LIP1 has a catalytic triad of Ser152-His253-Glu221 with a GGX-type oxyanion pocket, whereas LIP2 has Ser138-His249-Asp221 in its active site and a GX-type of oxyanion hole residues. However, LIP1 has a catalytic triad of Ser152-His253-Glu221 with an oxyanion pocket of GGX-type, whereas LIP2 has Ser138-His249-Asp221 in its active site and a GX-type of oxyanion hole residues. Our three-dimensional models of LIP1 and LIP2 complexed with a 3-hydroxyoctanoate dimer revealed the core α/β hydrolase-type domain with an exposed substrate binding pocket in LIP1 and an active-site capped with a closing lid domain in LIP2. The recombinant LIP1 was optimally active at 45°C and pH 9.0, and the activity improved in the presence of Ca^2+^. LIP2 exhibited maximum activity at 40°C and pH 8.0, and was unaffected by Ca^2+^. Despite different properties, the enzymes exhibited broadsubstrate specificity and were able to hydrolyze short chain length and medium chain length polyhydroxyalkanoates (PHAs), polylactic acid (PLA), and para-nitrophenyl (pNP) alkanoates. Gel Permeation Chromatography (GPC) analysis showed a decrease in the molecular weight of the polymers after incubation with LIP1 and LIP2. The enzymes also manifested some polymer-degrading activity on petroleum-based polymers such as poly(ε-caprolactone) (PCL) and polyethylene succinate (PES), suggesting that these enzymes could be useful for biodegradation of synthetic polyester plastics. The study will be the first report of the complete characterization of intracellular lipases from bacterial and/or *Pseudomonas* species. The lipases, LIP1 and LIP2 are different from other bacterial lipases/esterases in having broad substrate specificity for polyesters.

## Introduction

Lipase enzymes (E.C.3.1.1.3), found in diverse organisms such as animals, plants, fungi, and bacteria, catalyze the hydrolysis of ester bonds in triglycerides to free fatty acids and glycerol. They act at the interface between an insoluble phase of the substrate and an aqueous phase containing the enzyme (interfacial activation) (Reis et al., 2009). A unique property of true lipases that distinguishes them from other esterases is their enhanced activity at a nonpolar-aqueous interface (Fojan et al., 2000). Lipases show a wide-range of molecular sizes, substrate/positional specificities, and catalytic activities. They have a large number of nonpolar residues near the surface that cluster around the active site (Fojan et al., 2000). Many lipases exhibit a lid-like structure, and opening of the lid exposes the hydrophobic patches in the active site, leading to catalysis at the lipid-water interface, while the closed lid conformation is inaccessible to solvent (Derewenda et al., 1992; Fojan et al., 2000). Esterases, and a few lipases, do not have a lid structure (Uppenberg et al., 1994; Longhi et al., 1997).

Microbial lipases have been investigated for their multifold applications in organic synthesis, detergent formulations, oleochemistry and nutrition (Saxena et al., 2003). To date, many bacterial lipases have been identified, cloned, and characterized (Contesini et al., 2020). However, very limited information is available on the potential application of lipases (especially intracellular lipases) for degradation of polyester polymers/plastics. Extracellular lipases purified from *Bacillus subtilis*, *Pseudomonas aeruginosa*, *Pseudomonas alcaligenes*, and *Burkholderia cepacia* (former *Pseudomonas cepacia*) degrade polyesters of ω-hydroxyalkanoic acids such as poly(ε-caprolactone (PCL) and poly-4-hydroxyalkaonaote (P(4HB)) polymers (Jaeger et al., 1995). The extracellular lipase of a *Pseudomonas* sp. was also able to degrade PCL (Kim et al., 1992). Weak but detectable medium chain length polyhydroxylalkanoate (mcl-PHA) activity was observed with the extracellular lipases from *P. alcaligenes* and *P. aeruginosa* (Jaeger et al., 1995).

In a previous report from this lab(Sharma et al., 2019), the cell free culture supernatant of *Pseudomonas chlororaphis* PA23 was shown to hydrolyze the ester bonds of p-nitrophenyl fatty acid substrates and PHA polymers with different subunit composition. In the present study, two genes encoding intracellular lipases from *P. chlororaphis* PA23 were cloned and expressed in *E. coli* BL21(DE3) to study the enzyme properties and investigate their degradation activity toward various biodegradable polymers such as small chain length (scl-) and mcl-PHAs, polylactic acid (PLA), polyethylene succinate (PES), and PCL. This is the first report on the application testing of lipases as potential agents for the degradation of a wide-range of biobased and synthetic polyesters.

## Materials and Methods

### Bacterial Strains and Plasmid

*P. chlororaphis* PA23 is a plant growth-promoting bacterium isolated from the soybean rhizosphere that can suppress the growth of the fungal pathogen *Sclerotinia sclerotiorum* due to its ability to synthesize phenazine and pyrrolnitrine compounds (Savchuk and Fernando, 2004; Fernando et al., 2007). *Escherichia coli* DH5a and *E. coli* BL21 (DE3) were used as host cells for recombinant plasmid construction and protein expression, respectively. pET28a(+) (Novagen, Madison, USA) was used as an expression vector for the expression of the lipase genes, *lip1* and *lip2*. The PHA polymers used in the study were synthesized by *Pseudomonas putida* LS46 (International Depository Authority of Canada Accession Number 181110-03) (Sharma et al., 2011; Blunt et al., 2018).

### Chemicals, Media and Growth Conditions

Hexanoic acid, octanoic acid, nonanoic acid, decanoic acid, polylactic acid (PLA), polycaprolactone (PCL) and polyethylene succinate (PES) was purchased from Sigma Chemical Co. (St. Louis, MO). All other analytical grade products, chemicals and dyes were from either Sigma Chemical Co. (St. Louis, MO) or Fisher Scientific (Toronto, ON). To prepare the various PHA polymers, the *P. putida*LS46 was cultivated in Ramsay’s minimal medium (RMM) (Ramsay et al., 1994) at 30°C in 7 L Applicon Bioreactor with hexanoic, octanoic, nonanoic, or decanoic acid as the sole carbon source (Blunt et al., 2018). A copolymer of 3-hydroxybutyrate and 3-hydroxyvalrate [Poly (3-hydroxybutyrate-co-3-hydroxyvalerate, PHBV] was prepared from biodiesel fatty acid supplemented with 0.5% valeric acid using *Cuprividusnecator*H16 (Sharma et al., 2016). The monomer compositions of these PHA polymers were determined (Sharma et al., 2011) and are shown in Table 1. Luria Bertani (LB) broth and agar were used for the growth of *E. coli* DH5α and *E. coli* BL21 (DE3) cells.

**TABLE 1:** Composition of PHA polymers used in this study.

PHA^a^	Substrate	^a^Monomer composition of PHA (mol %)								
		3HB	3HV	3HHx	3HHp	3HO	3HN	3HD	3HDD	3HTD
PHBV	Glucose/Valerate	76.9	23.1	ND	ND	ND	ND	ND	ND	ND
PHHx	Hexanoic acid	ND	ND	82.4	ND	16.0	ND	1.6	ND	ND
PHO	Octanoic acid	ND	ND	6.5	ND	92.0	ND	1.5	ND	ND
PHN	Nonanoic acid	ND	ND	ND	18.7	ND	81.3	ND	ND	ND
PHD	Decanoic acid	ND	ND	5.2	ND	57.4	ND	37.0	0.4	ND

^a^PHBV polymer was synthesized by *C. nector* H16 and all the other PHA polymers were synthesized by *P. putida* LS46 (Sharma et al., 2016; Blunt et al., 2018). PHBV, Poly (3-hydroxybutyrate-co-3-hydroxyvalerate; PHHx, poly(3-hydroxyhexanoate); PHO, poly(3-hydroxyoctanoate); PHN, poly(3-hydroxynonanoate); PHD, poly(3-hydroxydecanoate); ND, not detected.

### DNA Manipulation and Plasmid Construction

The genomic DNA of *P. chlororaphis* PA23 was isolated from LB bacterial cultures using the Wizard DNA purification kit (Promega). DNA fragments containing genes encoding the lipase enzymes, *lip1* and *lip2* were generated separately by PCR amplification from of *P. chlororaphis* PA23 chromosomal DNA using phusion DNA polymerase (Thermo Scientific) and the primer combinations listedin Table 2. The nucleotide sequences of these genes were obtained from the whole genome sequence of *P. chlororaphis* PA23 from NCBI (Accession No. CP008696). Primers were designed to incorporate the restriction endonuclease sites NheI and HindIII at the 5′-ends of the forward and reverse primers, respectively.

**TABLE 2:** Primers used for the construction of recombinant lipase, *lip1* and *lip2* in pET28a vector.

Gene	Primers used
*lip1*	**FP:** ATA​GCT​AGC​ACA​ATG​ACC​CTT​CTC​TAT​CGC
	**RP:** ATA​AAG​CTT​ACG​GAT​GGA​TGA​CAG​GGC​CTG
*lip2*	**FP:** ATA​GCT​AGC​AGC​ACG​TTA​AGT​TGG​GTT​CGT
	**RP:** ATA​AAG​CTT​GGC​GGA​TTG​GCG​CGC​TTG​CAG

The PCR reaction was carried out in a Thermocycler (Biorad, USA) under defined conditions (initial denaturation at 98°C for 30 s followed by 34 cycles of 98°C for 10 s, 66°C for 30 s and 72°C for1 min, with final extension of 10 min at 72°C). The amplicon was cloned into the expression vector, pET-28a (+), and transformed into *E. coli DH5α*. Transformants were selected on LB kanamycin (50 μg/ml) plates, followed by screening of recombinants by colony PCR and restriction digestion to verify fall-out. The positive clones containing lipase genes, *lip1* and *lip2* wereconfirmed and verified for integration by sequencing at the nucleic acid sequencing facility Macrogen, Maryland, United States.

### Sequence Analysis and Homology Modeling

Amino acid sequence similarity searches for *P. chlororaphis* PA23 lipases, LIP1 (EY04_08410) and LIP2 (EY04_09635) were carried out using Basic Local Alignment Search Tool (BLAST) for proteins (http://blast.ncbi.nlm.nih.gov/Blast.cgi). The sequences used were retrieved from the Integrated Microbial Genome (IMG) (https://img.jgi.doe.gov) and/or NCBI (https://www.ncbi.nlm.nih.gov). Alignment of amino acid sequences was performed using Clustal Omega (https://www.ebi.ac.uk/Tools/msa/clustalo/) and Espript 3.0 (http://espript.ibcp.fr/ESPript/ESPript/). Amino acids contents were analyzed using the ProtParam tool (http://web.expasy.org/protparam/). Secondary structure predictions and other protein features were generated using the XtalPred server (http://ffas.burnham.org/XtalPred-cgi/xtal). The three-dimensional structures of LIP1 and LIP2 were modeled using SWISS-MODEL (Waterhouse et al., 2018). The putative thioesterase tm1040_2492 from *Silicibacter* sp. TM1040 (2PBL) for LIP1, and the monoglyceride lipase Rv0183 from *M. tuberculosis* (6EIC) (Aschauer et al., 2017, 2018) for LIP2 served as templates. A dimer of 3-hydroxyoctanoic acid (HO) was generated using Avogadro: an open-source molecular builder and visualization tool, version 1.2.0 (http://avogadro.cc/, Hanwellet al., 2012) and the universal force field (Rappe et al., 1992) was used for energy minimization. The dimer was docked to the LIP1 and LIP2 model using the Vinawizard in PyRx software (Dallakyan and Olson, 2015). Figures were generated using PyMOL (Molecular Graphics System, Version 2.5.2).

### Expression and Purification of Recombinant LIP1 and LIP2

The recombinant plasmids carrying the lipase gene (*pET28a-lip1* and *pET28a-lip2*) were isolated using the Geneaid plasmid isolation kit and introduced into *E. coli* BL21 (DE3) expression host. *E. coli* BL21 (DE3) cells harboring the recombinant plasmid were cultivated by inoculating 1% primary culture of the recombinant cells into 50 ml of LB broth supplemented with 50 μg/ml kanamycin. Cells were grown using an incubator shaker at 37°C until optical density of 0.6 at 600 nm (OD_600_) and induced with 0.6 mM isopropyl-1thio-β-D-galactopyranoside (IPTG) for 5 h. Subsequently, the expression levels of *lip1* and *lip2* were studied at various IPTG concentrations (0.4, 0.6, 0.8 and 1.0 mM), growth temperatures (37°C, 30 and 16°C) after induction, and incubation times (4, 8, and 16 h). The cells were harvested by centrifugation, and digested in chilled lysis buffer (25 mM Tris-HCl, 10 mM MgCl_2_, 100 mM NaCl, and 1 mg/ml lysozyme) in an ultrasound machine [10 cycles with 1 min pulse (10 s on/off)]. Recombinant enzymes were purified from the clear lysate (obtained after centrifugation at 10,000 × g for 30 min) by affinity chromotagraphy using HiTrap Ni^2+^-NTA resins (Qiagen) under nondenaturing condition according to the manufacturer’s instructions. Expression profiles and purity of recombinant proteins were analyzed by sodium dodecyl sulfate–polyacrylamide electrophoresis (SDS-PAGE).

### Qualitative and Quantitative Estimation of Depolymerase/Esterase Activity of LIP1 and LIP2

To determine the depolymerase activity of the recombinant enzymes, a homogeneous latex suspension of PHA polymers synthesized by *P. putida* LS46 from hexanoic (PHHx), octanoic (PHO), nonanoic (PNO), and decanoicacids (PHD) were prepared as described by Ramsay et al. (1994). Four volumes of a PHA solution in acetone (0.1%, w/v) were added dropwise to 1 volume of cold water (5–10°C) with stirring. The acetone was removed with speed vacuum concentrators to obtain a white colloidal suspension. PHA agar plates were prepared using 10 mg PHA polymer suspension and 1.5% (wt/vol) agar in 50 mM phosphate buffer (pH 7). The enzyme solution was dropped into 5-mm-diameter wells punched into the PHA agar plates and then incubated at 30°C for 48 h. The zone of clearance around the wells indicated the depolymerase activity. The fluorescent dye, Nile red (0.0005%) was added to the plates to reveal fluorescent clear halos around the wells when irradiated with UV light (350 nm) (Kouker and Jaeger, 1987).

The PHA depolymerase activity of the enzyme was determined by measuring the turbidity decrease of the PHA suspension at OD_650_ (Tamura et al., 2011). The reaction mixture contained 1 mg PHA latex, 50 mM phosphate buffer, pH 7 and 0.025 mg enzyme solution. The turbidity decrease was measured every 24 h for 96 h. The substrate control, PHA (without the enzyme) was taken in parallel. One unit of depolymerase activity by turbidimetric assay was determined as the amount of enzyme that can decrease the absorbance (OD_650_) by 1 absorbance unit per min. Alternatively, one unit of depolymerase activity (units_*PHA*_) was the amount of enzyme that hydrolyzes 1 μg of PHA in 1 min. Parallel PHA control samples (without the enzyme) were taken. All experiments were carried out in triplicate.

The esterase activity of lipases was determined as described by Schirmer et al. (1993) using p-nitrophenyl octanoate (PNPO) as substrate. The recombinant enzyme from crude cell lysate and/or the purified enzyme (0.025 mg) was used in a reaction with 3.9 ml PBS buffer and 100 µl substrate, PNPO (1 mM) at optimum temperature for 20 min. The reaction was stopped with 1 ml of 1 M sodium carbonate and the absorbance was recorded at OD_420_. The substrate control, PNPO (without the enzyme) was taken in parallel. One unit of PNPO esterase activity was measured as the amount of enzyme releasing 1 µmol of p-nitrophenol per min under optimal conditions. Total protein was determined by Bradford assay using Bovine serum albumin (BSA) as the standard protein (Bradford, 1996).

### Biophysical and Biochemical Properties, and Substrate Specificity of LIP1 and LIP2

To determine the effect of pH on the activity of LIP1 and LIP2, the substrate (PNPO, 1 mM) was dissolved separately in buffers with different pH values [sodium acetate buffer (50 mM, pH 4.0–5.0), sodium phosphate buffer (50 mM, pH 6.0–8.0) and glycine-NaOH buffer (50 mM, pH 9.0–10.0)], and then used in reaction mixtures with the appropriately diluted enzyme (0.025 mg) solutions prepared in the desired buffers. The effects of temperature on lipase activity were investigated by testing at different temperatures (25–55°C) for 20 min. The enzymes were exposed to different temperatures (30–60°C) for a period of 24 h to check their thermal stability. The effect of cations (chloride/sulfate salts), chelators (EDTA) and ionic and non-ionic detergents on the enzyme activities of LIP1 and LIP2 was studied by adding them to the reaction mixtures and incubating them for 20 min in 50 mM sodium phosphate buffer at their optimal pH and temperature conditions. The residual enzyme activities were determined.

The substrate specificity of the enzymes was estimated against various PHAs, PHBV, PLA, p-nitrophenylalkanoate substrates (pNP-acetate, pNP-butyrate, pNP-octanoate and pNP-decanoate), and petrochemical based polymers such as poly(ethylene succinate) [PES] and polycaprolactone (PCL) at 1 mg substrate concentration under optimal conditions for enzyme activity. V_max_ and K_m_ values were calculated using the Lineweaver-Burk linear regression plot. The purified enzymes, LIP1 and LIP2 were incubated with pNP octanoate substrate in concentrations ranging from 0.1–3.0 mM under optimal assay conditions.

### Gel Permeation Chromatography

The molecular weights of the PHAs before and after degradation with LIP1 and LIP2 were analyzed by gel permeation chromatography (GPC). The reaction mixture containing different 10 mg polymer suspension (PHB, PHHx, PHO, PHN, PHD, PLA, PCL, and PES) prepared according to Ramsay et al. (1994) was subjected to enzymatic hydrolysis with the recombinant lipases (2.5 mg) in separate experiments. The polymer substrates (without the enzyme) were kept as controls. After 96 h incubation at optimal temperature, the reaction mixtures and substrate controls were oven dried at 60°C. After cooling, samples were chloroformed to achieve a final sample concentration of 1.5 mg/ml and filtered with 0.45 mm PTFE. All test and control sample sets were examined by gel permeation chromatography (GPC) using a Waters Model 1515 solvent pump with a Waters Refractive Index detector (model 2414) and the Agilent PLgel MIXED-C column (7.5 mm id: 1.5 ml/min). Data were acquiredand analyzed using the Breeze 2 software (Waters Chromatography). The process was calibrated with Agilent Polystyrene EasiCal PS-1 standards (Agilent Technologies, Mississauga, Ontario, Canada). The mobile phase (HPLC grade chloroform) was run at a flow rate of 1 ml/min. The column temperature was set at 30°C and the sample injection volume was set at 20 µl. The method was integrated with the standards to obtain the peak-average molecular weight (MP), number-average molecular weight (Mn), weight-average molecular weight (Mw) and the polydispersity index (Mw/Mn).

## Results

### Cloning, Expression and Purification of LIP1 and LIP2

The genome sequence of *P. chlororaphis* PA23 is available at the NCBI (https://www.ncbi.nlm.nih.gov) and Integrated Microbial Genome (IMG) (https://img.jgi.doe.gov) (Genome Acc. No. CP008696). *P. chlororaphis* PA23 possess seven genes annotated as esterase/lipase (Table 3). Of these, three encode for intracellular lipases (EY04_08410, EY04_09635, EY04_02420), three encode a signal peptide and are extracellular lipases (EY04_21540, EY04_17885, EY04_32435), and one encodes for an intracellular PHA depolymerase (EY04_01535). In our previous study, the hydrolysis of the ester bonds of p-nitrophenyl-fatty acid substrates, and the ester bonds of PHA polymers of various subunit composition by the extracellular esterases/lipases was recorded in the culture media in which *P. chlororaphis* PA23 and *A. lwoffii* were grown (Sharma et al., 2019). In the present study, two genes encoding intracellular lipases were cloned and expressed in *E. coli* BL21(DE3) to compare the properties and investigate its biodegradability in comparison with various biodegradable polymers.

**TABLE 3:** Putative lipases present in the genome of *Pseudomonas chlororaphis* PA23.

Lipases	Accession number	Location	Gene size (bp)	Number of amino acids (aa)	Molecular weight (kDa)
LIP1	EY04_08410	Intracellular	828	275	29.7
LIP2	EY04_09635	Intracellular	834	277	30.2
LIP3	EY04_02420	Intracellular	891	296	32.5
LIP4	EY04_21540	Extracellular	942	315	34.0
LIP5	EY04_17885	Extracellular	1905	634	69.8
LIP6	EY04_32435	Extracellular	1911	636	70

**FIGURE 1:**
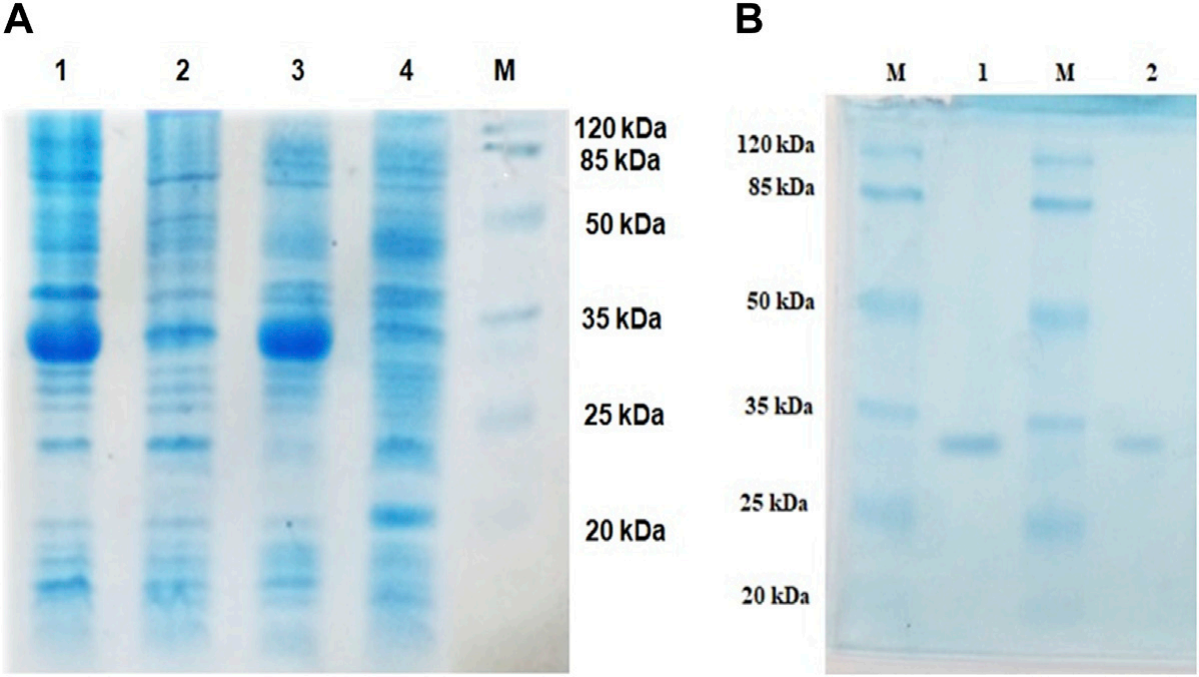
Expression and purification profiles (SDS-PAGE) of recombinant LIP1 and LIP2; **(A)** Cell lysates of *Escherichia coli* BL21 (DE3) transformed with *pET28a-lip1* and *pET28a-lip2* Lane 1, cell lysate of induced *E. coli* BL21 (DE3) carrying *lip1-pET28a* expressing LIP1; Lane 2, the lysate of the noninduced host cells containing *lip1-pET28a*; Lane 3, cell lysate of induced *E. coli* BL21 (DE3) harboring *lip2-pET28a* with an expression band of LIP2; Lane 4, cell lysate of the non induced *E. coli BL21* (DE3) with *lip2-pET28a plasmid*; Lane M, standard molecular weight marker (Thermo Fisher Scientific) **(B)** SDS-PAGE-purification profiles after His–Tag affinity chromatography: LIP1 (Lane 1) and LIP2 (Lane 2) on 12% SDS-PAGE; Lane M, standard molecular weight marker.

**FIGURE 2:**
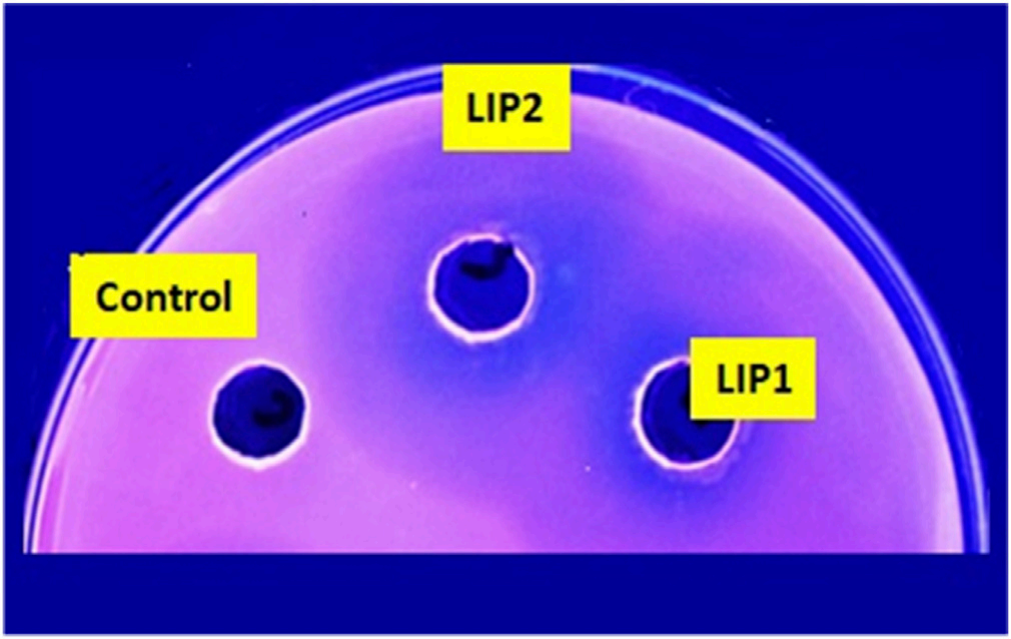
PHA agar plate assay for testing the depolymerase/esterase activity of LIP1 and LIP2. Qualitative mcl-PHA depolymerase activity of LIP1 and LIP2 in Nile red-PHO agar plate after UV irradiation (350 nm). Twenty (20) µg of purified LIP1 and LIP2 enzymes were added to the wells and incubated for 48 h. Substrate hydrolysis leads to the formation of fluorescent clear halos around the wells. The soluble crude extract of uninduced expression host *E. coli* BL21 (DE3) was used as control.

Lipase genes cloned into the pET28a(+) vector and expressed in *E. coli* BL21(DE3) showed optimal expression with 0.6 mM IPTG at 37°C in 5 h (Figure 1A). The recombinant proteins were purified to homogeneity. SDS-PAGE analysis of the expressed and purified enzymes revealed molecular masses of ∼30 kDa for LIP1 and LIP2, which correlated with the predicted molecular mass from its amino acid sequence (https://web.expasy.org/protparam) (Figures 1A,B). Qualitative assessment of the depolymerase activity of the purified enzyme was analyzed in a PHA agar plate with Nile red fluorescent dye (Figure 2). Compared to the control strain (the soluble crude extract of uninduced recombinant *E. coli* BL21 cells containing *lip1-pET28a(+)*/*lip2-pET28a(+)*, the purified enzymes were able to hydrolyze PHA latex as shown by the formation of fluorescent clear halos/zones around the wells after 48 h of incubation at 30°C, under UV irradiation (350 nm) (Figure 2).

### Sequence Analyses and Homology Modeling of LIP1 and LIP2

**FIGURE 3:**
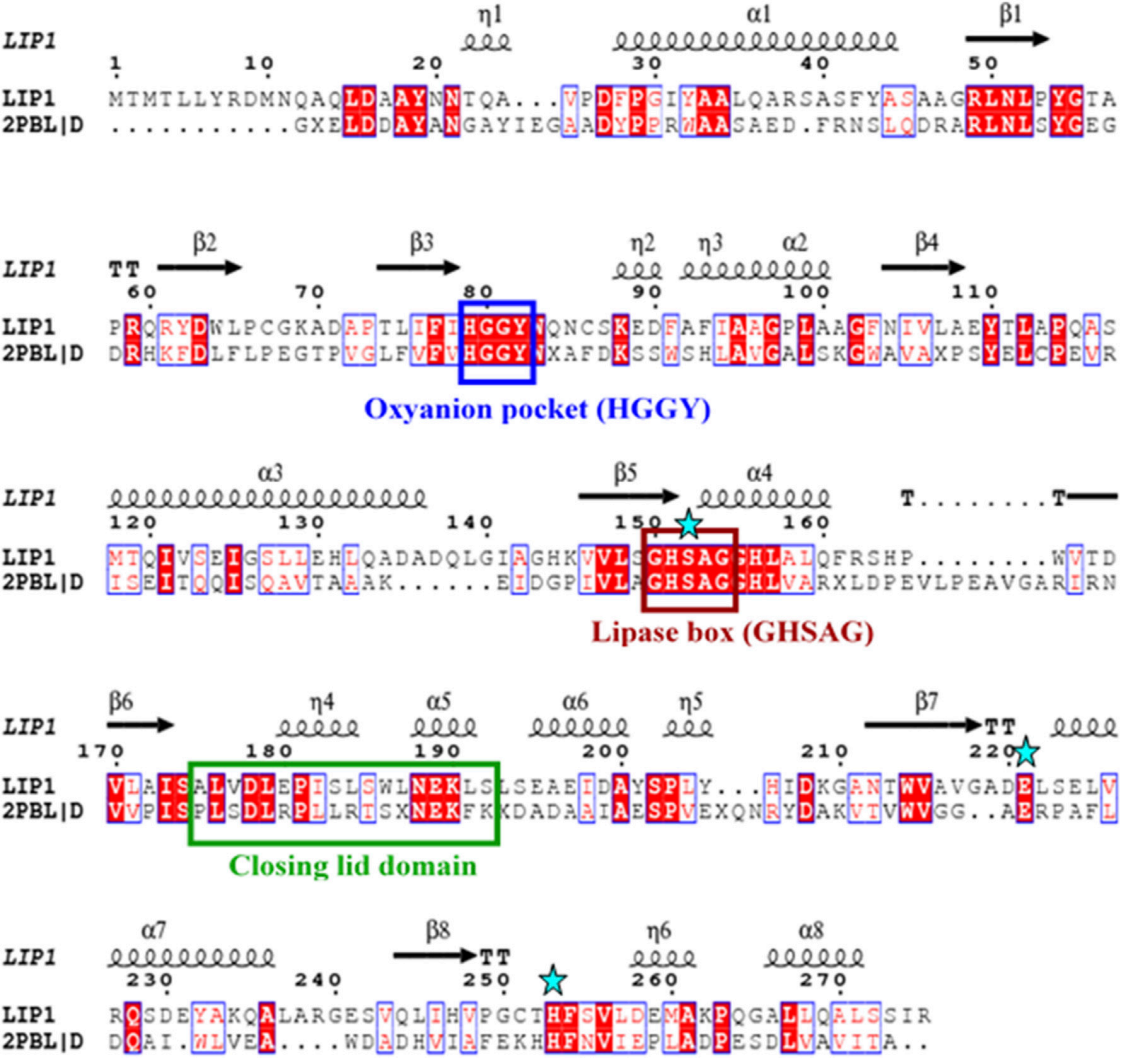
Amino acid sequence alignment of LIP1 with putative lipase/thioesterase from *Silicibacter* sp. Tm1040 (2PBL). The lipase consensus sequence is indicated by a brown box, the residuesof the oxyanion holes in a blue box, the domain of the closing lid in a green box and the residues of the catalytic triad (serine, glutamic acid and histidine) are highlighted by asterisks. α-helix and β-strands are shownat the top of the alignment.

**FIGURE 4:**
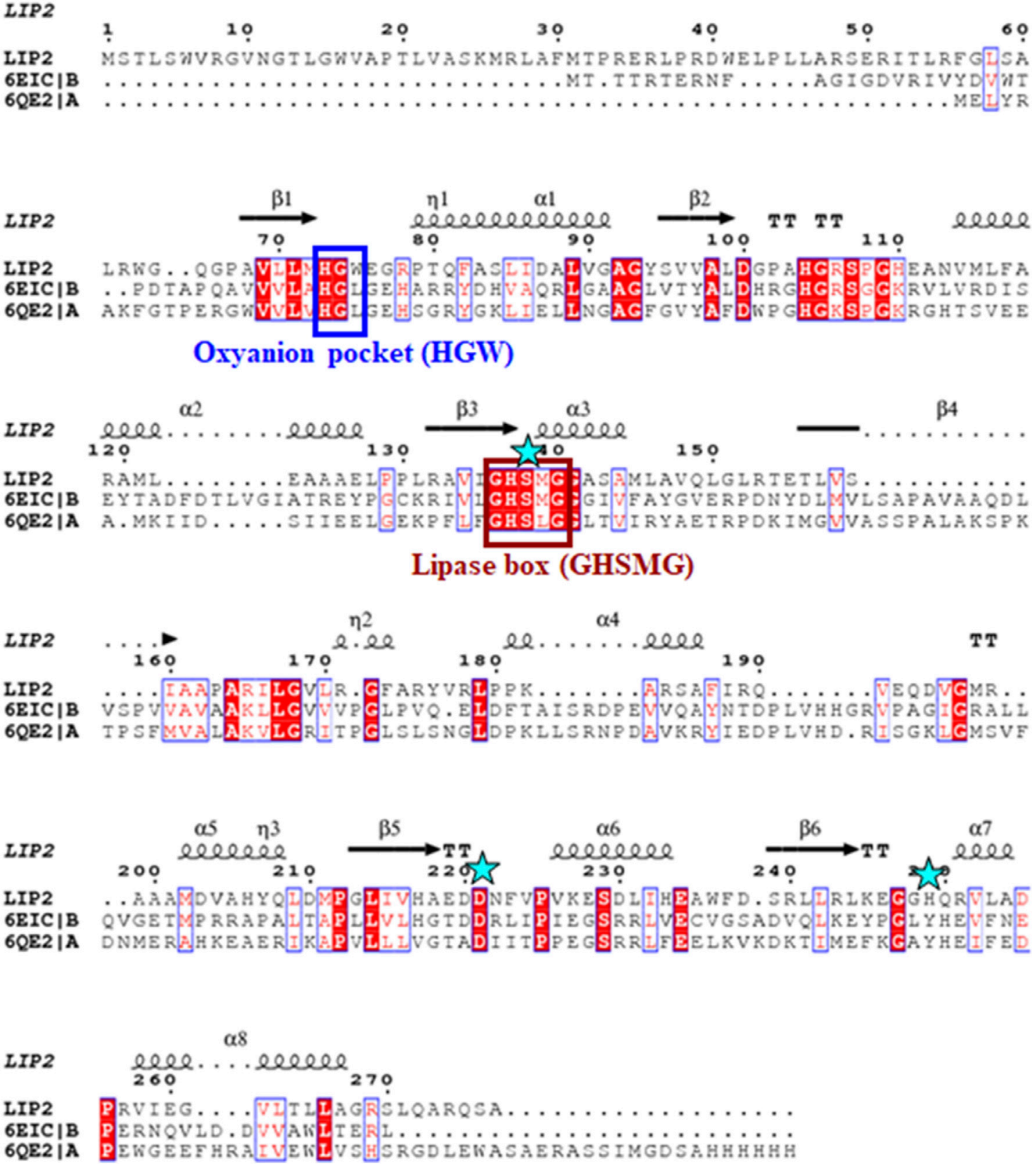
Amino acid sequence alignment of LIP2 with the monoglyceride lipase from *Mycobacterium tuberculosis* (6EIC) and monoacylglycerol lipase from *Paleococcus ferrophilus* (6QE2). The consensus sequence of lipase is shown in a brown box, the residues of the oxyanion hole are indicated by a blue box, and the residues of the catalytic triad (serine, aspartic acid and histidine) are highlighted by asterisks. The α-helix and β-strands are shown at the top of the alignment.

The *lip1* and *lip2* genes were found to encode enzymes with predicted molecular masses of 29.7 and 30.2 kDa, respectively. BLAST analyses of the deduced amino acid sequences (BLASTp) revealed that the enzymes, LIP1 and LIP2, belong to the lipase superfamily and have an α/β-hydrolase fold. Alignment of the amino acid sequences of LIP1 and LIP2 revealed very low sequence identity (15.39%). However, both enzymes have the conserved sequence regions including the lipase box (GX_1_SX_2_G) characteristic of the lipase/esterase family and the putative oxyanion hole residues (HGGY in LIP1; HGX in LIP2) in the catalytic domain (Figures 3, 4). The x_1_ sites in the lipase boxes of LIP1 and LIP2 are occupied by histidine (H151) and arginine (R137), whereas the x_2_ sites contain alanine (A153) and proline (P139), respectively. The enzymes possess a catalytic triad (serine-histidine-aspartic acid/glutamic acid) characteristic of other lipases. The catalytic triad of LIP1 has Ser^152^-His^253^-Glu^221^ (Figure 3), while the catalytic triad of LIP2 has Ser^138^-His^249^-Asp^221^ in its active site (Figure 4).

**FIGURE 5:**
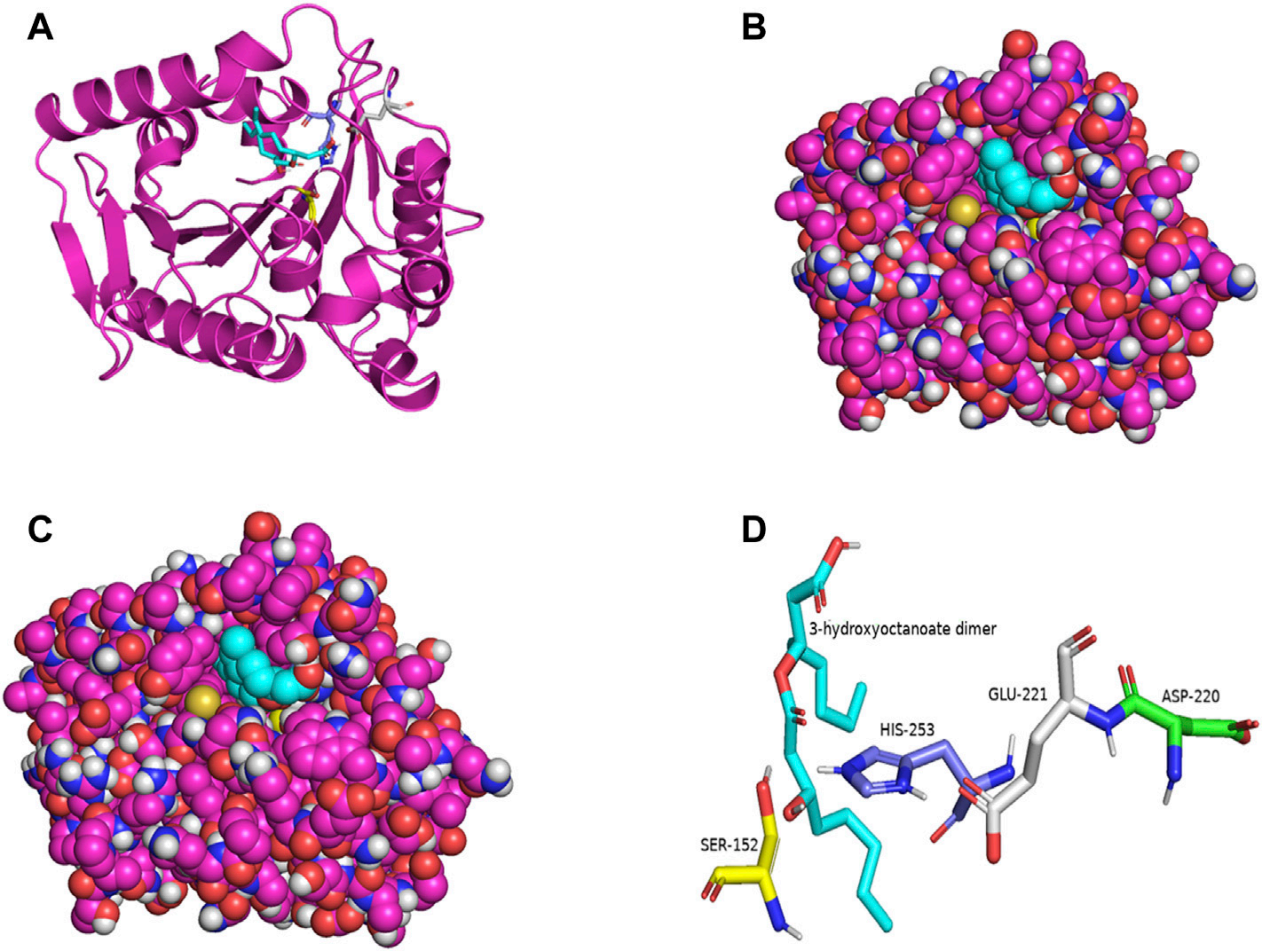
The three-dimensional structure of LIP1 generated by homology modelling. **(A)** Ribbon plot representation of the entire LIP1 scaffold with labelled residues of the catalytic triad Ser^152^ (yellow) His^253^ (slate) Glu^221^ (gray) in the binding pocket. The substrate 3-hydroxyoctanoate dimer (cyan) is computationally docked. **(B)** Atomic model of LIP1 with docked substrate in van der Waals representation. **(C)** Representation of the molecular surface of lipase LIP1 with the docked substrate (stick representation). **(D)** The closer view of the residues (stick model) of the catalytic triad around the substrate in the active site of LIP1, colored as in A, with Asp220 colored green. The structure is calculated using the putative esterase tm1040_2492 as template and SWISS-MODEL Program.

**FIGURE 6:**
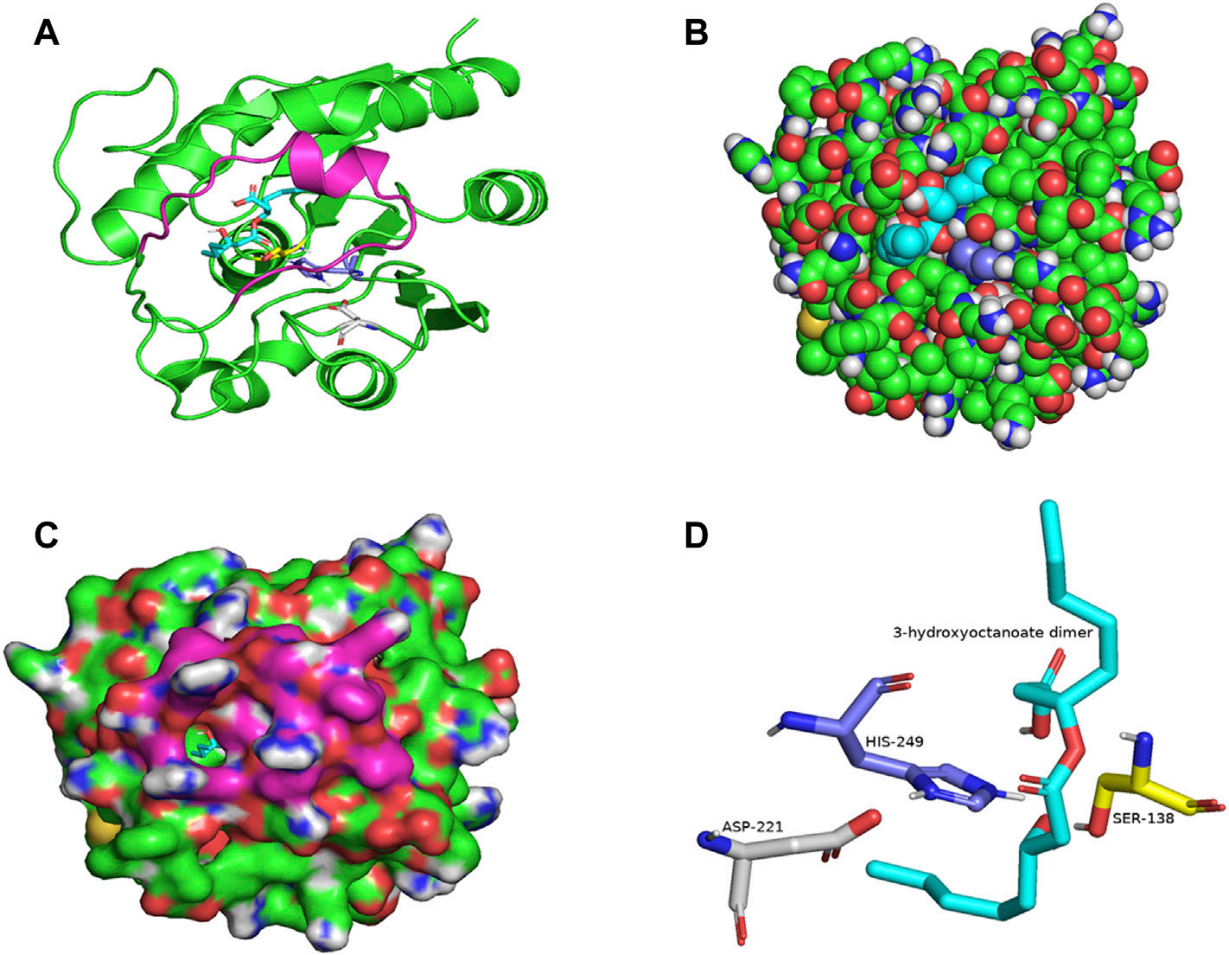
The three-dimensional structure of LIP2 generated by homology modelling. **(A)** Ribbon plot representation of the entire LIP2 scaffold with labelled residues of the catalytic triad Ser^138^ (yellow) His^249^ (slate) Asp^221^ (gray) in the binding pocket. The model also contains the lid domain (magenta) and the active site with the docked substrate 3-hydroxyoctanoate dimer (cyan). **(B)** Atomic model of LIP2 with docked substrate in van der Waals representation. Note that the lid domain is not shown to better visualize the binding pocket. **(C)** Illustration of the lipase LIP2 surface with the docked substrate (stick representation). **(D)** The closer view of the residues (stick model) of the catalytic triad around the substrate in the active site of LIP2, colored as in A. The structure is calculated using the monoglyceride lipase Rv0183 as template and SWISS-MODEL Program.

PSI-BLAST search for LIP1 and LIP2 in the Protein Data Bank revealed several bacterial lipases as close homologues. The putative esterase, tm1040_2492 from *Silicibacter* sp. TM1040 (2PBL) was chosen as a template for LIP1 (27% amino acid identity and 88% similarity), and monoglyceride lipase Rv0183 from *M. tuberculosis* (6EIC) was chosen for LIP2 (24% amino acid identity and 70% similarity). Figures 5A, 6A show the homology model of LIP1 and LIP2, respectively, with the substrate 3-hydroxyoctanoate. The core domain of LIP1 consists of an 8-parallel stranded β-sheet (except for strand-2 which is antiparallel), whereas LIP2 consists of a six-parallel stranded β-sheet. Multiple α-helices are located on both sides of the core β-sheet in each model. Figures 5B,C, 6B,C show the binding pocket of the substrate 3-hydroxyoctanoate dimer. Residues 175-192 of LIP2 are likely the closing lid domain of the substrate binding pocket, while the substrate binding pocket of LIP1 is exposed and lacks the closing lid (capping) domain. To better illustrate the substrate binding pocket, the closing lid domain is not shown in Figure 6B.

The catalytic triad of hydrolases and lipases usually involving closely spaced Ser-His-Asp/Glu residues is a hallmark of this enzyme class. We also identified the catalytic triad motif in both LIP1 both LIP2 and modeled their binding pockets with the substrate as shown in Figures 5D, 6D. In the LIP1 model, the distance between Oδ of Glu^221^ and Nδ of His^253^ is 3.1 Å. In comparison, in the LIP2 model, the Oδ of Asp^220^ is 12.4 Å away from His^253^, and therefore, Glu^221^ acts as the acid in the catalytic triad of LIP2. The binding energy for the substrate to LIP1 and LIP2 determined in the docking experiment was calculated to be −-6.4 kcal/mol and −6.1 kcal/mol, respectively. A negative free energy of binding indicates the substrate docking is spontaneous.

### Characterization of the Recombinant LIP1 and LIP2

**FIGURE 7:**
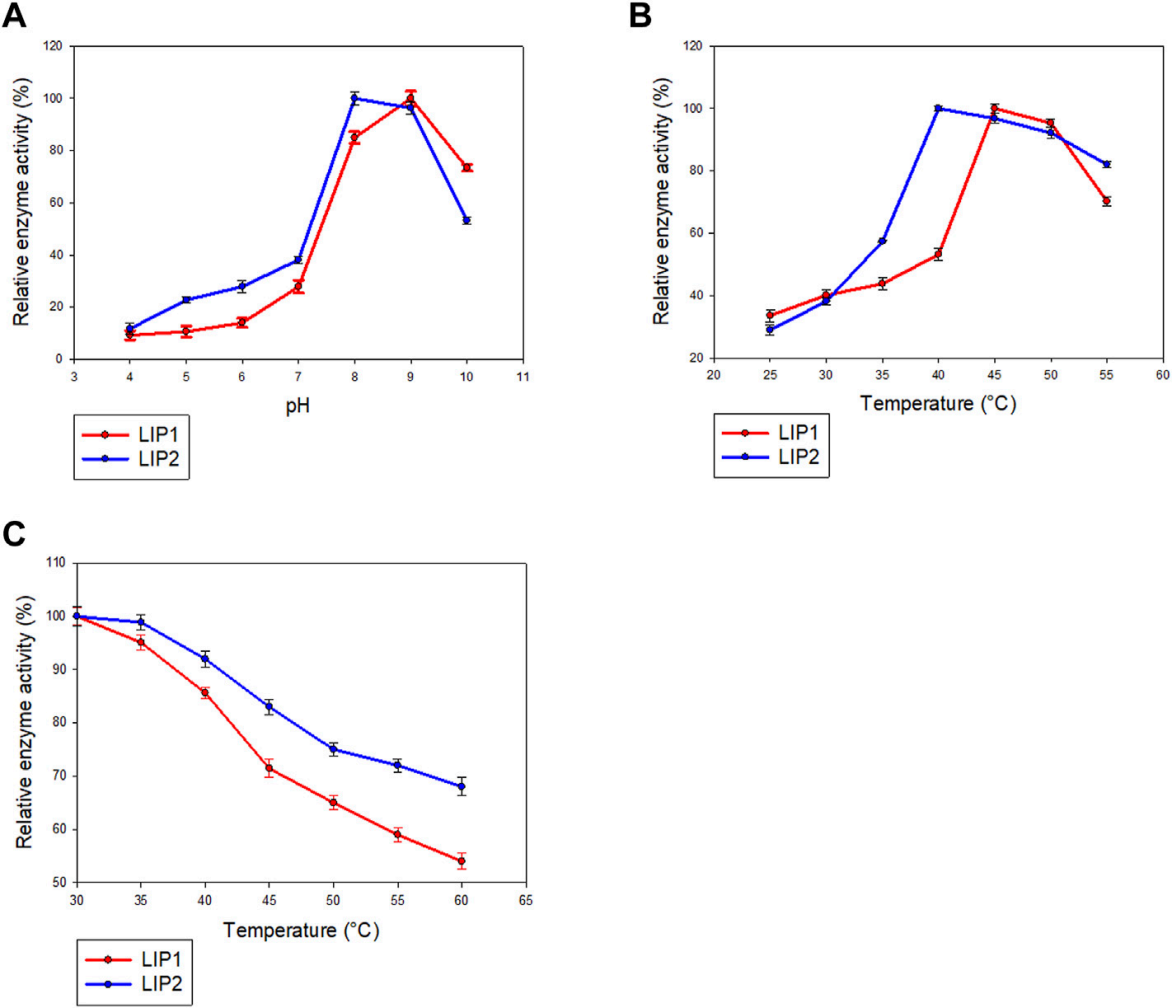
Determination of parameters of enzymatic activity of LIP1 and LIP2. Effects of **(A)** pH and **(B)** temperature on the activity of LIP1 (red line) and LIP2 (blue line). Recombinant LIP1 and LIP2 were optimally active at pH 9.0 and 8.0, respectively. The observed maximum activity was taken as100%. **(C)** Effect of temperature on the stability of LIP1 (red line) and LIP2 (blue line). The activity of the untreated enzyme (0.025 mg/ml) was set as 100%.

LIP1 and LIP2 were active over a wide pH range (pH 4.0–10.0) with optimal activity at pH 9.0 and pH 8.0, respectively (Figure 7A). LIP1 and LIP2 retained more than 50% of activity over a pH range from 8.0 to 10.0, butthe enzymesshowed less than 30% activity at acidic pH (4.0–6.0). LIP1 and LIP2 were optimally active at 45 and 40°C, respectively, and retained more than 50% activity when the reaction was carried out at 55°C (Figure 7B). Moreover, thermal inactivation experiments showed that the LIP1retained nearly 70% activity for 24 h at 45°C (Figure 7C). LIP2, on the other hand, was very stable and retained more than 80% of its activity for 24 h at 45°C. The enzymes were stable at 30°C for at least 2 months of incubation and retained nearly 100% of their activities for minimum 4 months at 4°C.Sequence analysis of LIP1 and LIP2 was performed to analyze thermophilic properties in relation to their amino acid sequence (Table 4). Enzyme kinetic studies using pNP-octanoate as substrate revealed that V_max_ and K_m_ for LIP1 were 769.23 μM mg^−1^ min^−1^ and 0.384 mM, respectively, whereas these values for LIP2 were 714.28 μM mg^−1^ min^−1^and 0.286 mM, respectively.

**TABLE 4:** Parametric comparison and amino acid composition analysis of LIP1 and LIP2.

Amino Acid	LIP1	LIP2
% Uncharged polar residues (Gln + Asn + Thr + Ser)	20.3	13.3
% Hydrophobic residues (Ala + Val + Ile + Leu)	37.1	38.6
% Arg	3.3	9.7
% Lys	2.5	1.4
% Pro	4.7	5.8
% Cys	1.1	0
% Arg/Lys	1.32	6.93

When the effect of cations and other compounds was examined (Table 5), it was found that 1 mM MgSO_4_, or 10 mM EDTA did not affect the enzyme activity, but the enzyme activity of LIP1 was increased by 50% in the presence of 1 mM CaCl_2_.The activity of LIP2 was not affected by the presence of EDTA and metal ions, indicating that it is not a metalloenzyme. The cations of magnesium, sodium and calcium are not required as cofactors for the enzyme activity. PMSF and ionic and nonionic detergents (SDS and Tween 80) inhibited the activities of both LIP1 and LIP2 (Table 5).

**TABLE 5:** Effects of various modulators on LIP1 and LIP2 activity.

Modulators/reagents	Final concentration	Relative activity (%)	
		LIP1	LIP2
EDTA	10 mM	98 ± 1.5	95 ± 3.1
CaCl_2_	1 mM	158 ± 3.5	82 ± 2.2
MgCl_2_	1 mM	79 ± 2.1	73 ± 1.2
NaCl	50 mM	110 ± 1.1	96 ± 0.5
PMSF	1 mM	69 ± 0.5	80 ± 3.3
Tween80	0.1%	30 ± 1.6	53 ± 0.9
SDS	5%	26 ± 1.0	19 ± 2.6
Enzyme activity (Without modulator)		100	100

a The values shown are the averages of three independent (biological) replicate experiments ± standard deviations.

### Substrate Specificity of LIP1 and LIP2

**FIGURE 8:**
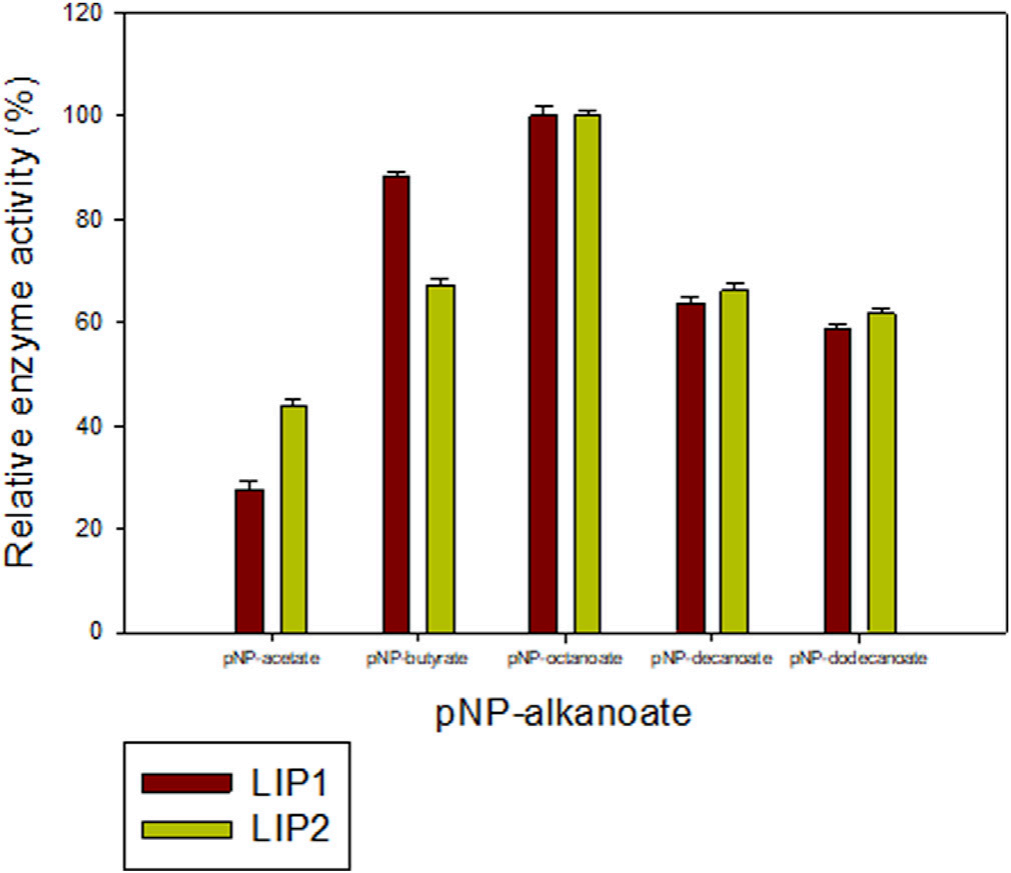
Determination of the activities of LIP1 and LIP2 towards different substrates. Substrate specificity of lipases LIP1 (brown bars) and LIP2 (green bars) from *P. chlororaphis* PA23. The reaction mixture contained 1 mM of various p-nitrophenyl(pNP) alkanoates and 0.025 mg of purified enzyme under optimal conditions of the enzymes. The maximum activity observed was taken as 100%. The 100% activity for LIP1 and LIP2 corresponded to 730.12 U mg^−1^ and 656.56 U mg^−1^for pNP-octanoate, respectively.

The lipase enzymes LIP1 and LIP2 showed broad substrate specificity. In particular, the enzymes demonstrated significant activity towards the ester bonds of pNP-alkanoates, which are the typical substrate for lipases (Figure 8). LIP1 and LIP2 hydrolyzed pNP-alkanoates ranging from shorter to longer side chains, with esterase activity highest for pNP-octanoate. The relative activity of LIP1 was significantly higher (88.24%) than that of LIP2 (67.23%) for pNP-butyrate. LIP1 and LIP2 were able to hydrolyze ester bonds of β-polyhydroxyalkanoates/PHA polymers prepared from valeric acid (PHBV), hexanoic acid (PHHx), octanoic acid (PHO), nonanoic acid (PHN), and decanoic acid (PHD), as shown by the turbidimetric assays with different polymers (Table 6). The order of substrate (PHA) preference for the LIP1 was PHO > PHN > PHD > PHHx > PHBV while for LIP2 the order of substrate preference was PHO > PHN > PHHx > PHBV > PHD. With PHO as substrate, LIP1 and LIP2 had specific activity of 360.12 units_*PHA*_ mg^−1^ and 301.72 units_*PHA*_ mg^−1^ of protein, respectively. Taken together, these results indicate that the enzyme has a broad substrate spectrum ranging from scl-and mcl-PHAs to pNP-alkanoates (C4 to C10). Remarkably, in addition to PHA polymers, the enzymes showed significant activity towards PLA and were able to hydrolyze synthetic polyester plastics such as PCL and PES (Table 6).

**TABLE 6:** Substrate specificity of the purified LIP1 and LIP2.

Substrate	Relative activity (%)^a^	
	LIP1	LIP2
**PHA polymers**		
PHBV	74.35 ± 2.2	63.45 ± 3.1
PHHx	79.31 ± 5.5	74 ± 2.6
PHO	100	100
PHN	92.33 ± 3.2	82.84 ± 4.3
PHD	83.19 ± 4.9	59.31 ± 3.6
**Other biodegradable/petrochemical-based polymers**		
polylactic acid (PLA)	42.32 ± 1.2	47.89 ± 4.3
poly(ɛ-caprolactone) (PCL)	32.96 ± 3.2	38.11 ± 1.4
poly(ethylene succinate) (PES)	17.09 ± 2.3	14.66 ± 3.7

a The values shown are the averages of three independent (biological) replicate experiments ± standard deviations. The pure enzyme (0.025 mg) was assayed with the indicated substrates at a final concentration of 1 mg, in all cases for 96 h. Polymer without the enzyme was taken as control. The maximum activity observed was taken as 100%. 100% activity corresponded to 360.12 U mg^−1,^ in LIP1 and 301.72 U mg^−1,^ in LIP2.

### Analysis of Degradation Products

Gel Permeation Chromatography (GPC) analyses of the polymer samples before and after enzyme treatment revealed significant degradation of PHAs, PLA, and the petrochemical-based polymers, PCL and PES by both LIP1 and LIP2. The chromatograms for each set of experiments with PHBV, PHHx, PHO, PHN, PHD, PLA, PCL, and PES showed a decrease in molecular weight in the polymer samples treated with LIP1 and LIP2 relative to the untreated/control samples (Supplementary Figures S1–S8). Degradation was achieved for scl-PHA (PHBV), mcl-PHAs (PHHx, PHO, PHN, PHD), PLA, PCL and PES for both LIP1 and LIP2 after 96 h of enzyme treatment. Separate chromatograms for each of the samples are included in Supplementary Figures S1–S8. The quantitative results of GPC analysis revealed significant changes in Mw, Mn, MP, polydispersity and other related parameters (Supplementary Table S1) for the polymers treated with LIP1 and LIP2 compared to the untreated samples/control.

## Discussion

Our previous report showed that the cell free culture supernatant of *P. chlororaphis* PA23 was shown to hydrolyze the ester bonds of p-nitrophenyl fatty acid substrates and PHA polymers of various subunit composition (Sharma et al., 2019). In the present study, two genes encoding intracellular lipases (designated here as LIP1, EY04_08410 and LIP2, EY04_09635) from *P. chlororaphis* PA23 were cloned and expressed in *E. coli* BL21 (DE3). Analysis of the primary structures of the LIP1 and LIP2 enzymes revealed no significant overall sequence homology between them. However, the lipase consensus sequence motif GX_1_SX_2_G was found with the X_1_ occupied by histidine and arginine residues in LIP1 and LIP2, respectively. In lipases and esterases, the X_1_ residue is commonly occupied by a polar residue and X_2_ is variable (Schirmer et al., 1993). In contrast, PHA depolymerases have a hydrophobic residue at position X_1_ (Knoll et al., 2009; Gangoiti et al., 2012). Another conserved sequence region (HGGY- in LIP1 and HGX-in LIP2) that appears to resemble the oxyanion hole consensus sequence in lipases was observed. Depending on the amino acids involved in the formation of the oxyanion hole, lipolytic enzymes are divided into two classes, the GGGX (X can be F, L or Y) and the GX type (Fischer and Pleiss, 2003; Pleiss et al., 2000). Thus, LIP1 belongs to the GGGX type, in which the first G is replaced by H, whereas LIP2 belongs to the GX class.

Lipases are generally highly variable in size, and sequence similarity among them is limited to short regions located around active-site residues. Arpigny and Jaeger (1999) suggested that bacterial lipases can be grouped into eight classes based on their conserved amino acid sequence regions and biochemical properties. However, the three-dimensional structures of lipases share a common folding motif in their cores, known as α/β hydrolase-fold, which is characteristic of hydrolase and/or esterase family enzymes (Ollis et al., 1992). The general α/β-hydrolase-fold consists of an eight-stranded, mostly parallel β sheet flanked by six α helices, with a catalytic triad (Ser/Asp/Cys-His-Asp/Glu); LIP1 has eight β sheets with Ser^152^-His^253^-Glu^221^ in its active site, while LIP2 has six β sheets and Ser^138^-His^249^-Asp^221^as a catalytic triad.

The enzymes exhibited different pH and temperature optima/stability but were similar to the lipase from *Pseudomonas aeruginosa* MB 5001, which showed maximum esterase activity at pH 8.0 and 55°C (Chartrain et al., 1993). The lipases of psychrotrophic *Pseudomonas fluorescens* strain AFT 36(Fox and Stepaniak, 1983) and *Pseudomonas* sp. (Choo et al., 1998) were found to be stable between pH 6 and 9. The lipases from *Pseudomonas* sp. and *P. fluorescens* strain AFT 36 were found to be stable below 60°C (Fox and Stepaniak, 1983; Dong et al., 1999). PueB lipase from *Pseudomonas chlororaphis* (Howard et al., 2001) and lipase from*Pseudomonas* sp. (Adams and Brawely, 1981) exhibited stability at 100°C. The thermal stability of LIP2 was relatively higher than that of LIP1 as inferred from the thermal in activation studies. Sequence analysis of LIP1 and LIP2 also revealed relatively high thermostability for LIP2 compared with LIP1. LIP2 has few uncharged polar residues, a high arginine/lysine ratio and a high proline content (Table 4), the sequence based parameters which contribute to its increased thermal stability, as defined by Szilagyi and Zavodsky, 2000).

The stimulatory effect of Ca^2+^ on the LIP1 activity was similar to that observed for lipases from *Pseudomonas putida* 3SK (Lee and Rhee, 1994), *P. aeruginosa* EF2 (Gilbert et al., 1991) and *Pseudomonas* sp. 7323 (Zhang and Zeng, 2008). It has been suggested that the calcium ion is primarily involved in removal of fatty acids formed as insoluble calcium soaps during hydrolysis, thus causing a change in the interfacial substrate-water ration that is favorable for enzyme action (Brockman, 1994). The addition of calcium ion could also lead to better alignment on the substrate molecule or the formation of the calcium salts of fatty acids (Macrae and Hammond, 1985; Godtfredsen, 1990). Esterase activity was not affected by the presence of EDTA and metal ions in LIP2, suggesting that it is not a metalloenzyme and/or metal ions are not required as cofactors for enzyme activity. Similar observations were made for the lipases of *Trichosporon asteroides* strain LP005 (Dharmsthiti and Ammaranond, 1996) and *Aspergillus terreus* (Yadav et al., 1998). PMSF causes sulfonylation of the Oγ atom of the active-site serine residue, thereby obliterating the catalytic region and leading to irreversible inhibition of the enzyme activity (Sharma and Radha Kishan 2011). The inhibition of enzyme activity by PMSF in the enzymes was comparable to that of PueB lipase from *Pseudomonas chlororaphis*, which showed 50% inhibition with 1 mM PMSF (Howard et al., 2001). Inhibition of LIP1 and LIP2 activities by ionic and nonionic detergents, SDS and Tween 80 indicates the presence of a hydrophobic region in the catalytic center and/or a change in the active configuration of the enzyme. Inhibition of enzyme activity by SDS has been reported for lipase from *Pseudomonas putida* 3SK (Lee and Rhee, 1994), while *Thermomyces lanuginosus* lipase stays active at high SDS concentrations at alkaline pH (Rasmussen et al., 2022).

Modelling the three-dimensional structures of LIP1 and LIP2 complexed with a 3-hydroxyoctanoate dimer revealed the core domain to be of the α/β hydrolase-type with an active site capped with a closing lid domain in LIP2 and an exposed substrate binding pocket in LIP1. Most lipases function at an organic-aqueous interfaces facilitated by a mobile subdomain flap or lid located over the active site (Brocca et al., 2003; Khan et al., 2017). The lid protects the active site and is therefore responsible for catalytic activity (Barbe et al., 2009). In aqueous media, the lid is mostly closed, whereas in the presence of a hydrophobic layer, the open form is likely to be the predominant structure. The lids of lipases are amphipathic structures; in the closed from, their hydrophobic side is directed toward the catalytic pocket, while the hydrophilic side faces the solvent (Brocca et al., 2003). When the enzyme changes to the open conformation, the hydrophobic side becomes exposed so that it contributes to the substrate-binding region (Yang and Lowe, 2000). The amphipathic nature of the lid and its specific amino acid sequence are important factors contributing to the enzyme activity and specificity of lipases (Holmquist et al., 1995). However, some lipases do not have a lid structure such as CalB, the lipase from *Candida Antarctica* B (Uppenberg et al., 1994).

Interestingly, LIP1 and LIP2 showed broad substrate specificity with the ability to degrade pNP-alkanoates (C2-C12), scl- (C4) and mcl-PHAs (C6-C10), PLA, PCL and PES. The PueB lipase from *Pseudomonas chlororaphis* was also reported that have esterase activity with various pNP-alkanoates such as pNP-acetate, pNP-propionate and pNP-butyrate (Howard et al., 2001). While the LIP1 and LIP2 enzymes exhibited maximum activity with pNP-octanoate a different relative activity with the polyesters was found. LIP1 showed high relative activity to pNP-butyrate (C4) compared to LIP2. LIP1 has a comparatively high substrate preference for medium-chain PHAs which can be attribute to its high relative activity for PHO, PHN and PHD. In contrast, LIP2 has comparatively low degradation activity for PHD. The difference in substrate specificity maybe related to the different amino acid composition of the oxyanion pocket (GGX-type oxyanion in LIP1 versus GX-type in LIP2) and the active site configuration of the enzymes with different binding energy with the substrates.

PES is a poly(alkenedicarboxylate), while PCL is an aliphatic polyester composed of repeating ω-hydroxyalkanoate units. These polymers are mainly used in thermoplastic polyurethanes among many other applications (Fatma et al., 2014). The cell-free supernatant of yeast culture, *Pseudozyma japonica*-Y7-09 showed significant ability to degrade PCL (Fatma et al., 2014). The extracellular lipases of *P. aeruginosa* revealed the degradation of aromatic–aliphatic polyesters and polyesteramides (Wilkes and Aristilde, 2017). However, none of the studies determined the degradation activity of lipases for PES (Santos et al., 2013). In contrast, both LIP1 and LIP2 were able to hydrolyze both PES and PCL to some extent. An intracellular mcl-PHA depolymerase of *Pseudomonas putida* LS46 also showed detectable activity towards PES (Mohanan et al., 2020). The Gel Permeation Chromatography revealed a significant degradation of the polymers by LIP1 and LIP2, as shown from the decrease in molecular weights of the polymers treated with the enzymes (18–40% molecular weight loss of PHAs), which clearly shows its high degradation potential compared to the known lipases/esterases. PHA treated with *Bacillus subtilis* lipase reported the molecular weight decrease of 21% (Kanmani et al., 2016). The major distinguishing feature of LIP1 and LIP2 from other lipases available in literature (Table 7) is their broad substrate specificity. In addition to PHAs, PLA and p-nitrophenyl esters of fatty acids, the enzymes were able to hydrolyze synthetic polyesters, PES and PCL. Further, majority of the studies have been done using extracellular lipases (Table 7); this report study will be the first to provide complete characterization of intracellular lipases from bacterial and/or *Pseudomonas* species for biodegradation of polymers.

**TABLE 7:** Degradation of polyesters by bacterial lipases/esterases.

Bacterial source	Lipases/Esterases	Polyesters hydrolyzed/Degradation ability of the enzymes	Polyesters not hydrolyzed	Method Used	Activity	References
*Pseudomonas* sp.	Extracellular lipase	Polycaprolactone	—	Weight loss	—	Kim et al. (1992)
*Streptomyces rochei*, *Pseudomonas fragi*, *Pseudomonas* sp.	Extracellular lipases (commercial )	Poly(3-hydroxypropionate), Polycaprolactone	Poly(3-hydroxybutyrate)	Weight loss of polyester films	0.3–1.0 mg weight loss	Mukai et al. (1993)
			Poly(4-hydroxybutyrate)			
			Poly(5-hydroxyvalerate)			
			Poly(6-hydroxyhexanoate)			
*Pseudomonas fluorescens*	Extracellular lipase (commercial)	Poly(3-hydroxypropionate)	Poly(3-hydroxybutyrate), Poly(5-hydroxyvalerate)	Weight loss of PHA films	0.4–0.8 mg weight loss	Mukai et al. (1993)
		Poly(4-hydroxybutyrate)				
		Poly(6-hydroxyhexanoate)				
*Bacillus subtilis*, *Pseudomonas aeruginosa*, *Pseudomonas alcaligenes*, *Burkholderia cepacia* (former *Pseudomonas cepacia*)	Extracellular lipases	pNP-palmitate, Poly(6-hydroxyhexanoate) Poly(4-hydroxybutyrate), Polycaprolactone	Poly(3-hydroxybutyrate) Poly(3-hydroxyalkanoates), Polylactic acid	Turbidimetric assay, Rhodamine agar plate assay	*B. subtilis* lipase:	Jaeger et al. (1995)
					0.2 × 10^3^ U/mg (pNPP)	
					6000 U/mg (PCL)	
					*P. aeruginosa* lipase:	
					52 × 10^3^ U/mg (pNPP)	
					1.8 × 10^6^ U/mg (PCL)	
					*P. alcaligenes* lipase:	
					8 × 10^3^ U/mg (pNPP)	
					140,000 U/mg (PCL)	
					*B. cepacia* lipase:	
					0.5 × 10^3^ U/mg (pNPP)	
					40,000 U/mg (PCL)	
*P. fluorescens* GK13	Extracellular esterase	pNPP	Polyhydroxyalkanoates, Polycaprolactone, Polylactic acid	Turbidimetric assay	0.4 × 10^3^ U/mg (pNPP)	Jaeger et al. (1995)
				Rhodamine agar plate assay		
*Pseudomonas* sp. AKS2	Extracellular esterase and lipase	Low density polyethylene	-	Viability test in biofilm	-	Tribedi et al. (2015)
*Bacillus subtilis*	Extracellular lipase	pNPP, Polyhydroxyalkanoates	-	Molecular weight decrease by GPC	21.3% molecular weight decrease, 28.3% weight loss, 273.65 U/mg (pNPP)	Kanmani et al. (2016)
				FTIR		
				NMR		
				DSC		
*Geobacillus zalihae*	Extracellular lipase	Poly(3-hydroxybutyrate)	-	Poly(3-hydroxybutyrate) agar plate	Clear zone around the colony	Mohamed et al. (2017)
*Pseudomonas chlororaphis* PA23-63-1	Extracellular lipases and esterases mix	pNP-alkanoate	Polycaprolactone, Polyethylene sulfonate	PHA agar plate assay, Turbidimetric assay, Weight loss of PHA films	4.5% weight loss, 997.7 U/mg (pNPO), 722 U/mg (PHO), Clear zone of PHA hydrolysis	Sharma et al. (2019)
		Poly (3-hydroxybutyrate-co-3-hydroxyvalerate)				
		Poly(3-hydroxyhexanoate)				
		Poly(3-hydroxyoctanoate)				
		Poly(3-hydroxynonanoate)				
		Poly(3-hydroxydecanoate)				
*Pseudomonas chlororaphis* PA23	Intracellular lipases, LIP1 and LIP2	Poly (3-hydroxybutyrate-co-3-hydroxyvalerate	-	Nile blue agar plate assay, Turbidimetric assay, Molecular weight decrease by GPC	18–40% molecular weight decrease, Clear zone of PHA hydrolysis, 769.23 U mg^−1^ in LIP1 (pNPO), 714.28 U mg^−1^ in LIP2 (pNPO), 360.12 U mg^−1^ in LIP1 (PHO), 301.72 U mg^−1^ in LIP2 (PHO)	This study
		Poly(3-hydroxyhexanoate)				
		Poly(3-hydroxyoctanoate)				
		Poly(3-hydroxynonanoate)				
		Poly(3-hydroxydecanoate)				
		Polylactic acid				
		Polycaprolactone				
		Polyethylene sulfonate				

^a^pNP, paranitrophenyl; pNPP, paranitrophenyl palmitate; pNPO, paranitrophenyl octanoate; PHA, polyhydroxyalkanoate; PHBV, Poly (3-hydroxybutyrate-co-3-hydroxyvalerate; PHO, Poly(3-hydroxyoctanoate); PLA, polylactic acid; PCL, polycaprolactone; PES, polyethylene succinate; LDPE, low density polyethylene; GPC, gel permeation chromatography; FTIR, fourier transform infrared; NMR, nuclear magnetic resonance; DSC, Differential Scanning Calorimetry.

## Conclusions and Future Prospects

The present study demonstrated the characteristic features and structure-function relationship of the two lipases, LIP1 and LIP2 of *P. chlororaphis* PA23. It provides further evidence for the remarkable versatility of lipases with respect of their potential application for the degradation of various polyesters, including pNP-alkanoates, PHAs, PLA, and to some extent, the petrochemical-based polymers, PCL and PES. Our data suggests that LIP1 and LIP2 lipases can contribute to the biodegradation of various polyesters. Thus, the increasing demand for biodegradation agents for plastics could be satisfied by the use of microbial lipases. Directed evolution strategies/mutagenesis to improve the degradation potential and/or substrate specificity of the enzymes (LIP1 and LIP2) so as to degrade other synthetic polyesters such as PE/PET as well as analysis and comparison of the degradation behavior of the wild type and mutant enzymes will be our future target. Further, future studies to determine the high-resolution 3D structure of these enzymes will shed more light in elucidating their specific interactions with polyesters and other substrates of interest. Furthermore, this structural information will be also useful in engineering binding pockets of these enzymes either by classical mutagenesis of AI-assisted directed evolution to study and/or improve or even redesign their degradation potential towards different polyesters.

## Data Availability

The original contributions presented in the study are included in the article/Supplementary Material, further inquiries can be directed to the corresponding author.
