# Supplementary material for: Characterization of Polymer Degrading Lipases, LIP1 and LIP2 From Pseudomonas chlororaphis PA23
Source: Front Bioeng Biotechnol. 2022 Apr 20;10:854298. doi: 10.3389/fbioe.2022.854298 (PMC9065602; doi:10.3389/fbioe.2022.854298)
Supplement: Supplementary file 1 [file DataSheet1.PDF]

Supplementary Material

Characterization of polymer degrading lipases, LIP1 and LIP2 from *Pseudomonas chlororaphis* PA23

Nisha Mohanan[1], Chun Hin Wong[2], Nediljko Budisa[2] and David B. Levin[1]*

[1]Department of Biosystems Engineering, University of Manitoba, Winnipeg, Manitoba, R3T 5V6, Canada

[2]Department of Chemistry, University of Manitoba, 144 Dysart Rd., Winnipeg, Manitoba, R3T 2N2, Canada

*Correspondence

Prof. David B. Levin

davidlevin@umanitoba.ca

Table of contents

1. Supplementary Figures S1-S8 (Gel Permeation Chromatograms) 2 - 9

2. Supplementary Table 1 10-11

A

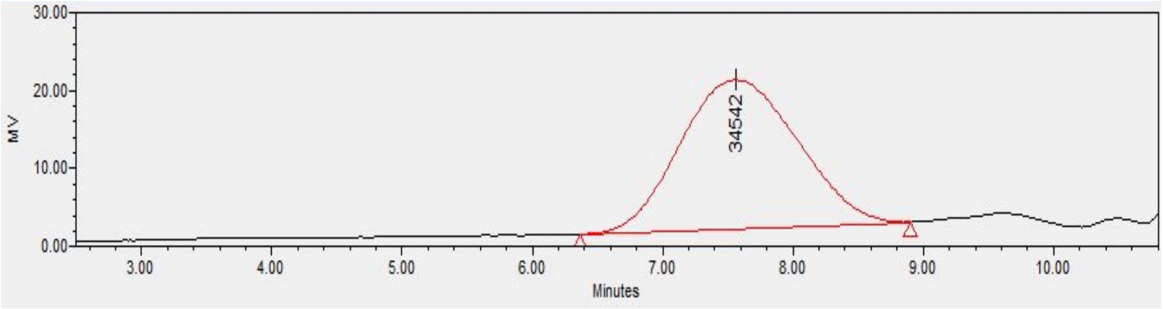

B

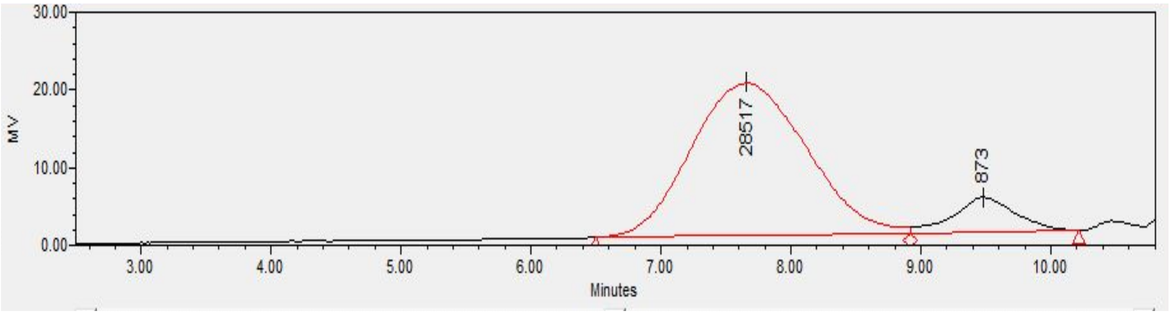

C

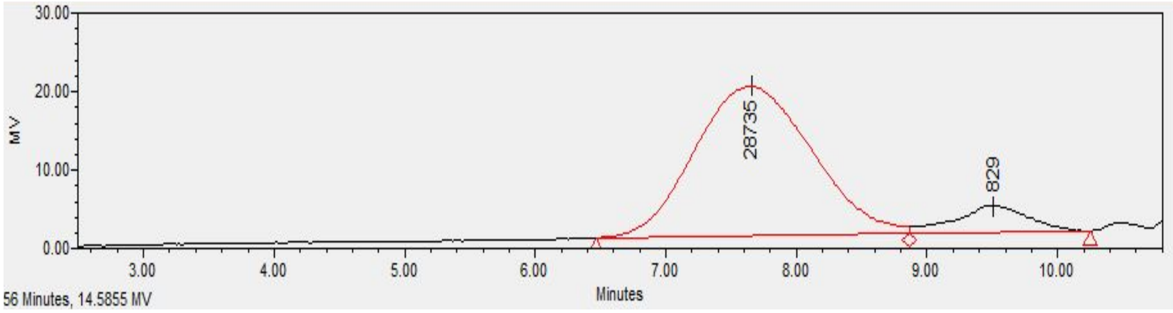

Supplementary Figure S1. Gel Permeation Chromatograms of the A) PHBV polymer before treatment with LIP1/LIP2; B) PHBV polymer after treatment with LIP1; and C) PHBV polymer after treatment with LIP2.

A

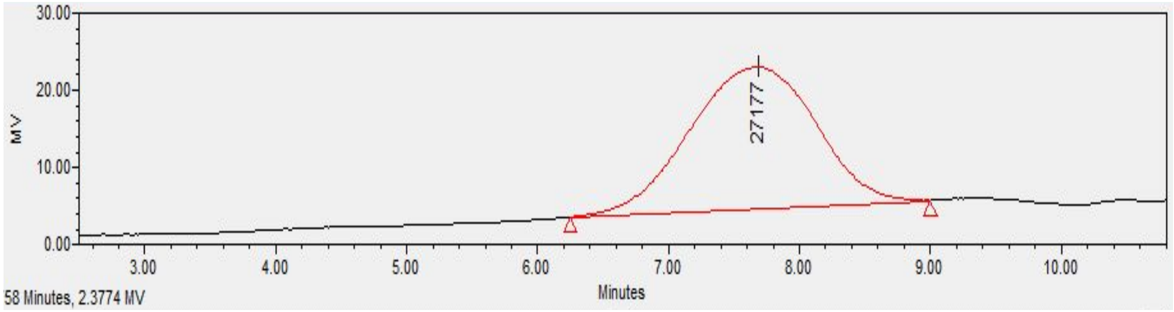

B

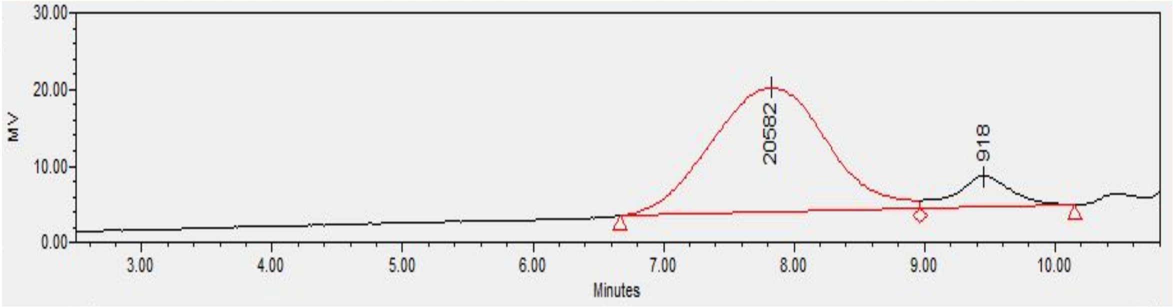

C

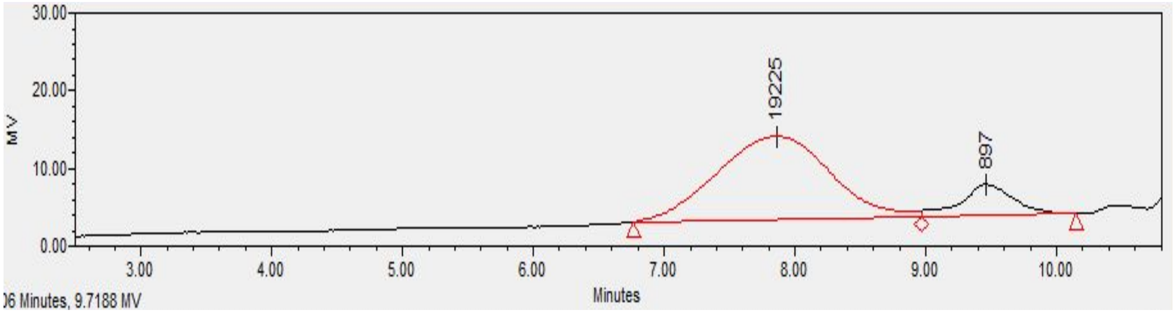

Supplementary Figure S2. Gel Permeation Chromatogram of A) the PHHx polymer before treatment with LIP1/LIP2; B) PHHx polymer after treatment with LIP1; and C) PHHx polymer after treatment with LIP2.

A

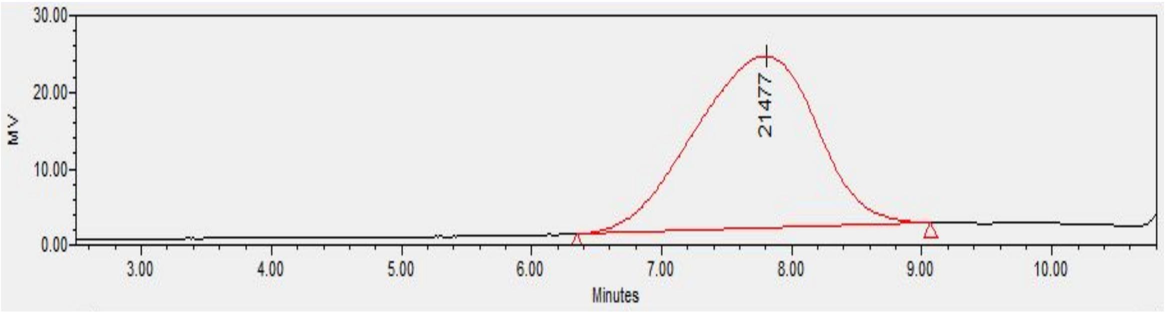

B

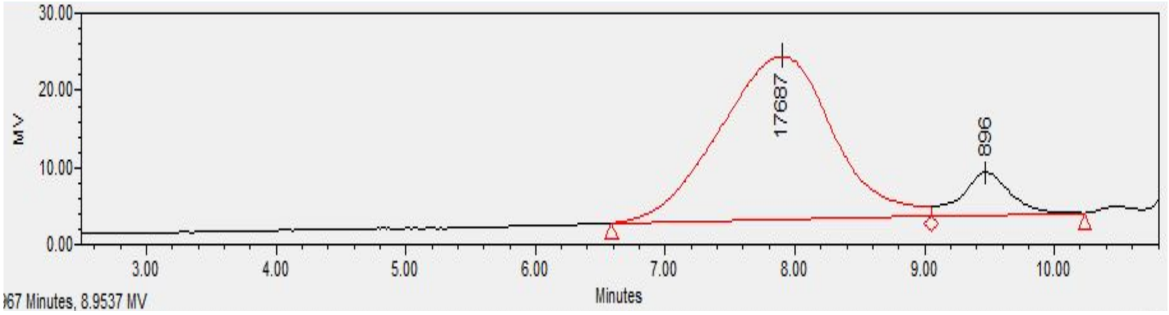

C

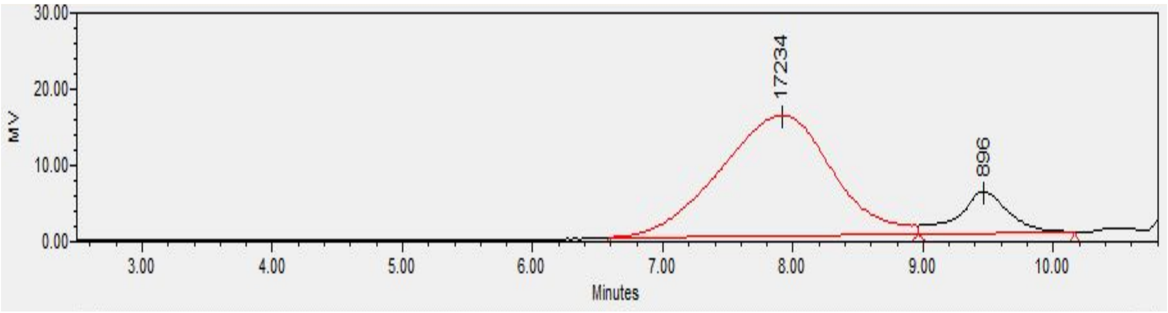

Supplementary Figure S3. Gel Permeation Chromatogram of A) the PHO polymer before treatment with LIP1/LIP2; B) PHO polymer after treatment with LIP1; and C) PHO polymer after treatment with LIP2.

A

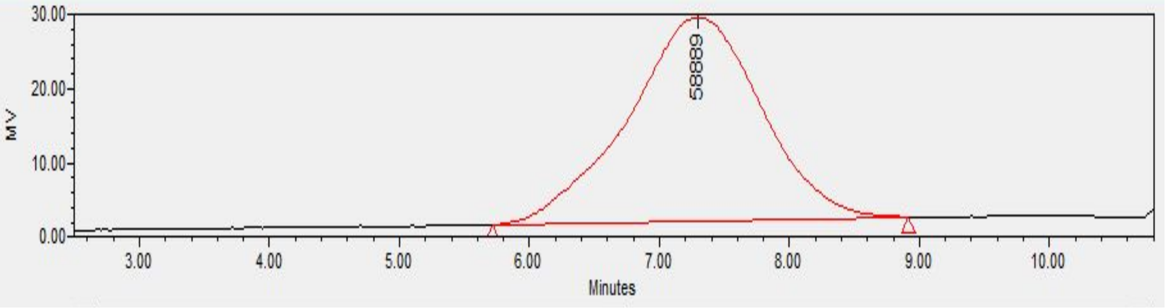

B

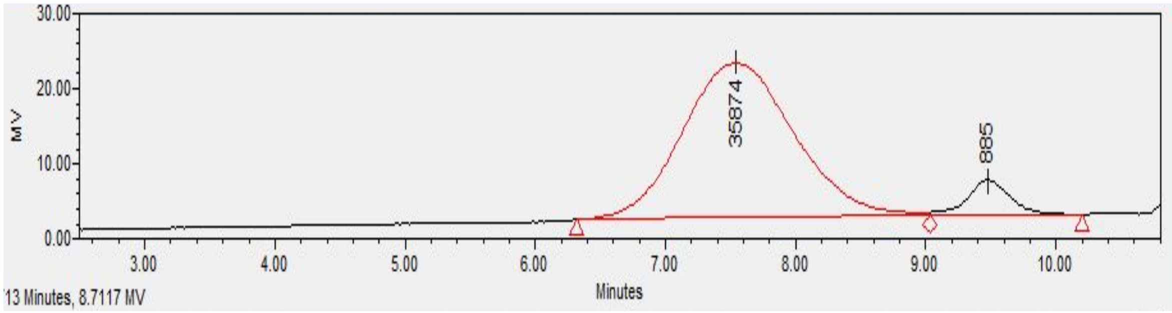

C

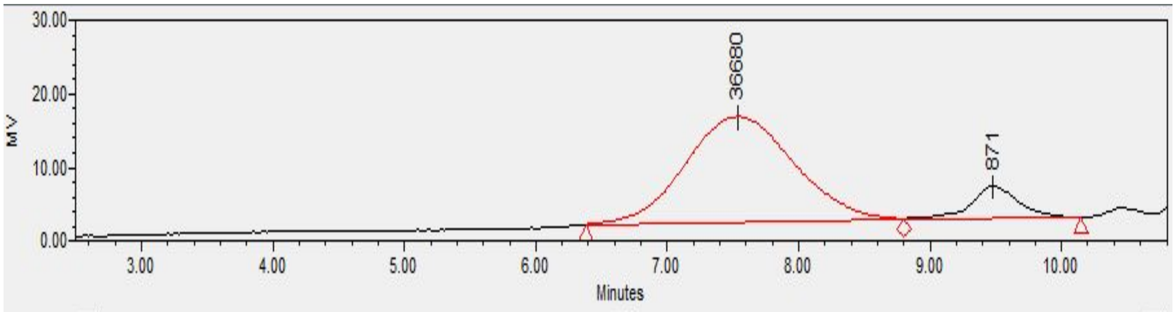

Supplementary Figure S4. Gel Permeation Chromatogram of A) the PHN polymer before treatment with LIP1/LIP2; B) PHN polymer after treatment with LIP1; and C) PHN polymer after treatment with LIP2.

A

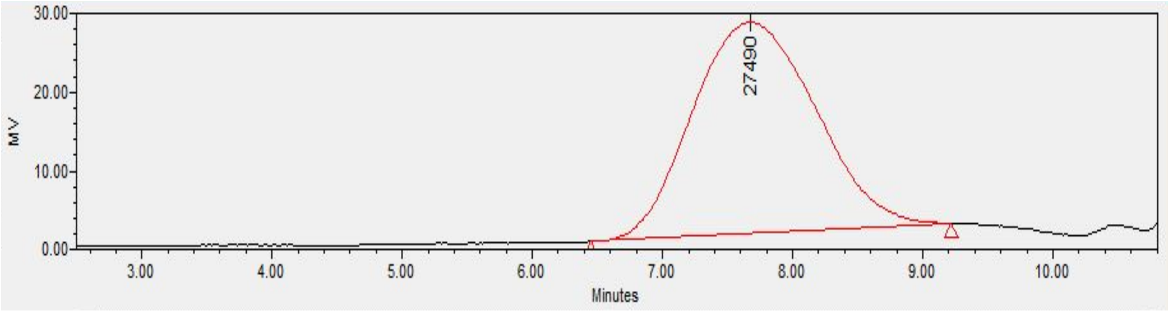

B

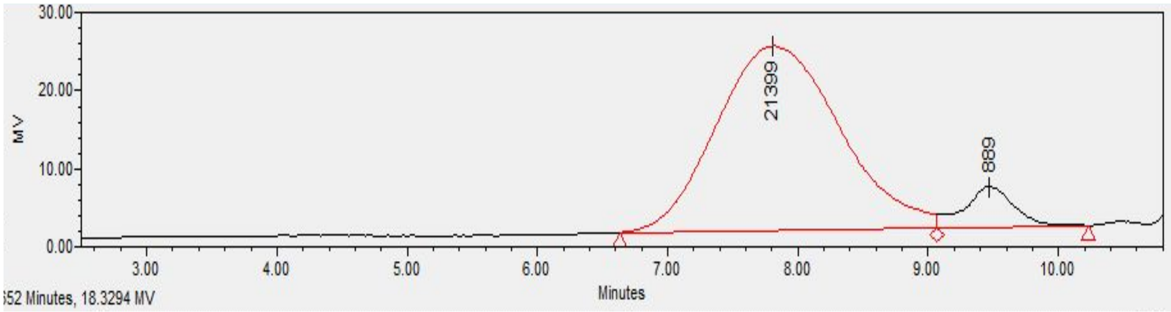

C

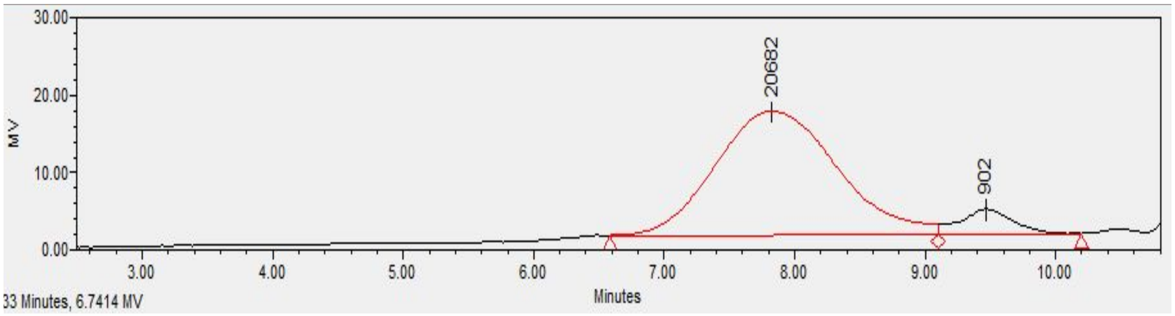

Supplementary Figure S5. Gel Permeation Chromatogram of A) the PHD polymer before treatment with LIP1/LIP2; B) PHD polymer after treatment with LIP1; and C) PHD polymer after treatment with LIP2.

A

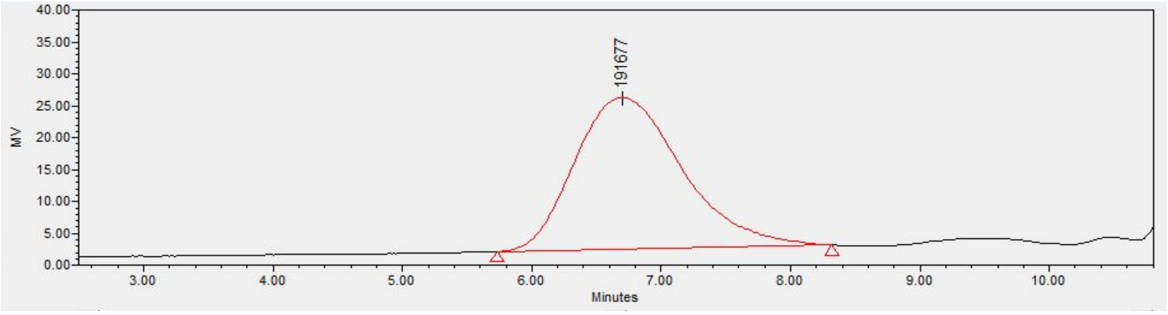

B

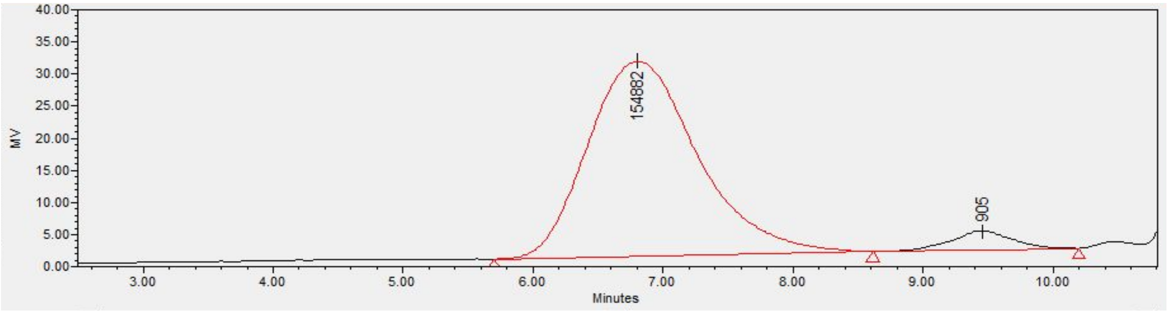

C

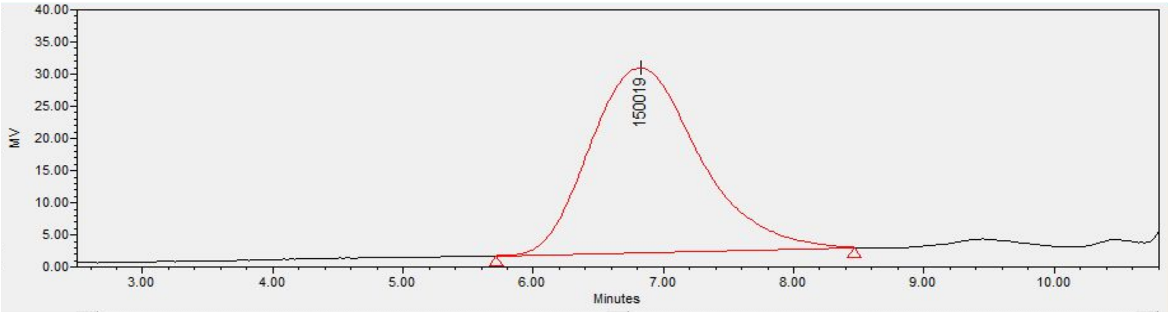

Supplementary Figure S6. Gel Permeation Chromatogram of A) the PLA polymer before treatment with LIP1/LIP2; B) PLA polymer after treatment with LIP1; and C) PLA polymer after treatment with LIP2.

A

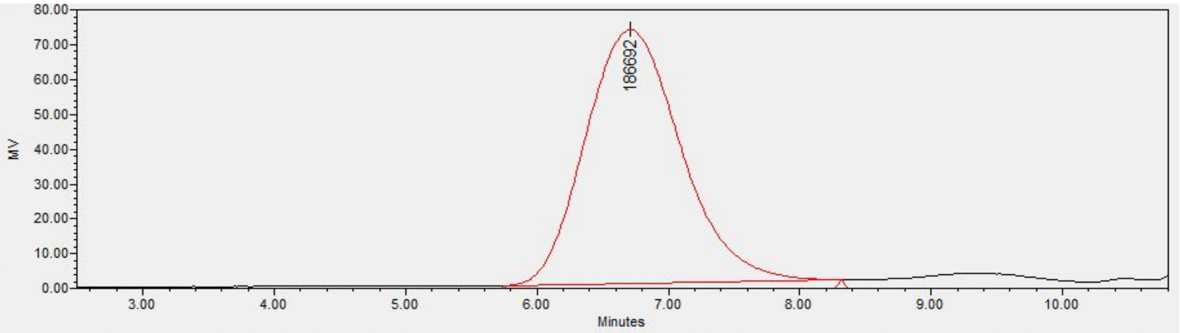

B

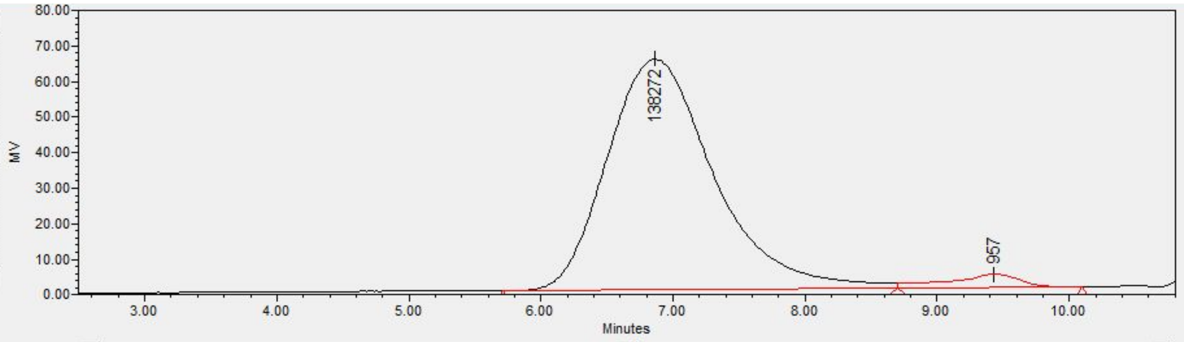

C

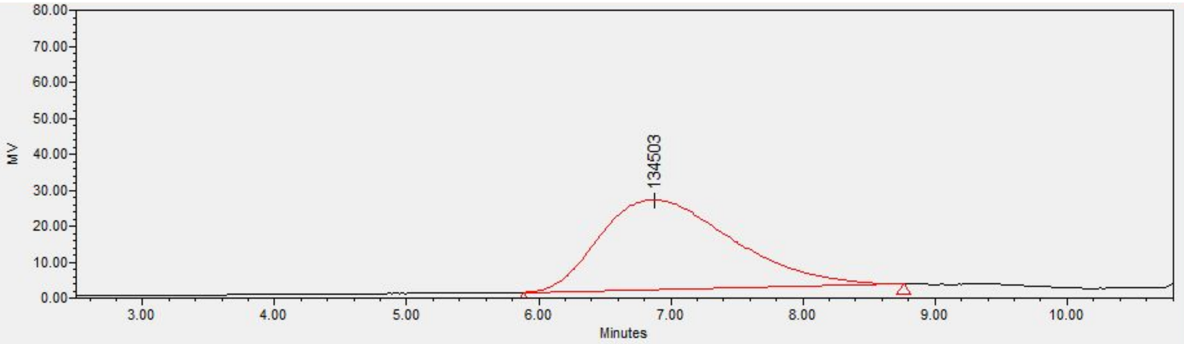

Supplementary Figure S7. Gel Permeation Chromatogram of A) the PCL polymer before treatment with LIP1/LIP2; B) PCL polymer after treatment with LIP1; and C) PCL polymer after treatment with LIP2.

A

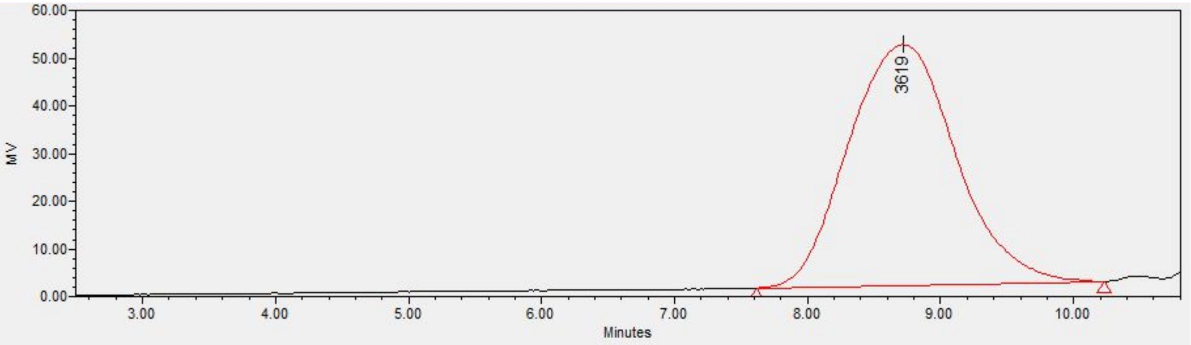

B

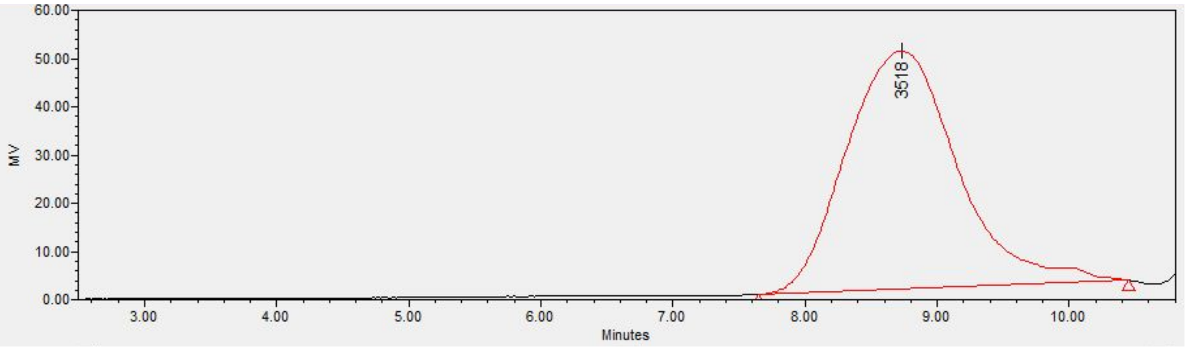

C

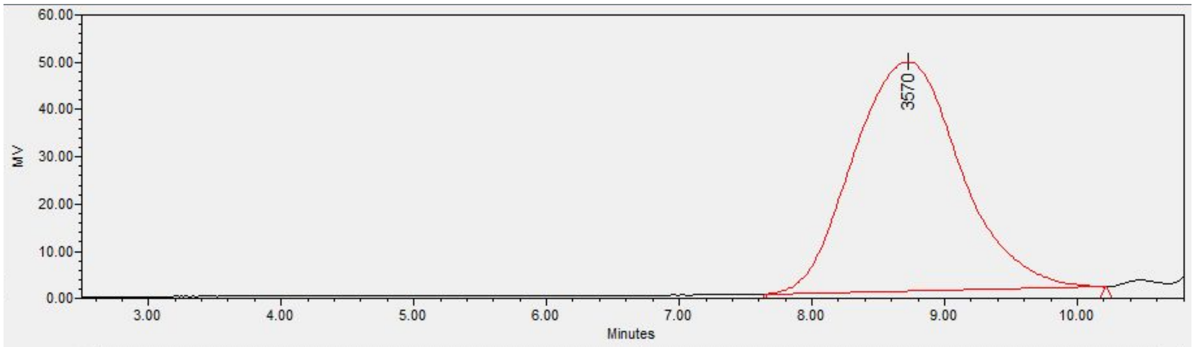

Supplementary Figure S8. Gel Permeation Chromatogram of A) the PES polymer before treatment with LIP1/LIP2; B) PES polymer after treatment with LIP1; and C) PES polymer after treatment with LIP2.

Supplementary Table S1. Quantitative results of GPC analysis depicting changes in the polymer properties after treatment with LIP1 and LIP2 relative to untreated polymers (control).

Polymer	Test	Peaks	Retention time (Minutes)	Mn (Da)	Mw (Da)	MP (Da)	Mz (Da)	Polydispersity (Mw/ Mn)	% Area	Height
**PHBV**	Control	Peak 1	7.564	23416	42700	34542	70858	1.82	100	19040
	After reaction with LIP1	Peak 1	7.661	19076	35263	28517	58138	1.85	89.17	19555
		Peak 2	9.480			873			10.83	4456
	After reaction with LIP2	Peak 1	7.657	19260	36146	28735	60847	1.88	89.86	18994
		Peak 2	9.507			829			10.14	3430
**PHHx**	Control	Peak 1	7.685	21305	42212	27177	80663	1.98	100	18267
	After reaction with LIP1	Peak 1	7.825	14969	28009	20582	47594	1.87	89.16	16244
		Peak 2	9.454			918			10.84	4177
	After reaction with LIP2	Peak 1	7.859	14562	26400	19225	43730	1.81	85.00	10693
		Peak 2	9.466			896			15.00	4008
**PHO**	Control	Peak 1	7.803	18931	36805	21477	69468	1.94	100	22399
	After reaction with LIP1	Peak 1	7.901	13883	26767	17687	48070	1.93	89.75	21126
		Peak 2	9.466			896			10.25	5597
	After reaction with LIP2	Peak 1	7.914	13985	26194	17234	47466	1.87	86.86	15702
		Peak 2	9.466			896			13.14	5403
**PHN**	Control	Peak 1	7.296	40489	101608	58889	249310	2.51	100	27411
	After reaction with LIP1	Peak 1	7.545	23012	44312	35874	75067	1.93	91.95	20611
		Peak 2	9.473			885			8.05	4674
	After reaction with LIP2	Peak 1	7.534	25094	44594	36680	73728	1.78	87.55	14330
		Peak 2	9.481			871			12.45	4476
**PHD**	Control	Peak 1	7.679	17584	34145	27490	57727	1.94	100.00	26827
	After reaction with LIP1	Peak 1	7.805	12847	25877	21399	45131	2.01	91.97	23509

		Peak 2	9.471			889			8.03	5168
	After reaction with LIP2	Peak 1	7.822	12472	25224	20682	44565	2.02	91.94	15969
		Peak 2	9.463			902			8.06	3206
**PLA**	Control	Peak 1	6.698	111284	209736	191677	329504	1.88	100	23760
	After reaction with LIP1	Peak 1	6.806	87701	174933	154882	282213	1.99	94.86	30349
		Peak 2	9.461			905			5.14	2996
	After reaction with LIP2	Peak 1	6.822	89405	170903	150019	272131	1.91	100	28635
**PCL**	Control	Peak 1	6.711	130101	210551	186692	306961	1.62	100	72723
	After reaction with LIP1	Peak 1	6.863	69777	150431	138272	236333	2.16	96.15	64863
		Peak 2	9.432			957			3.85	3818
	After reaction with LIP2	Peak 1	6.877	59427	142106	134503	244037	2.39	100	24883
**PES**	Control	Peak 1	8.719	2685	4453	3619	6684	1.66	100	50502
	After reaction with LIP1	Peak 1	8.734	2161	4254	3518	6536	1.97	100	49303
	After reaction with LIP2	Peak 1	8.726	2631	4397	3570	6598	1.67	100	48468

Da, Daltons; Mn, polymer average mass number; Mw, polymer average weight number; MP, peak average molecular weight; Mz, higher average molecular weight
